# Emergence of methicillin resistance predates the clinical use of antibiotics

**DOI:** 10.1038/s41586-021-04265-w

**Published:** 2022-01-05

**Authors:** Jesper Larsen, Claire L. Raisen, Xiaoliang Ba, Nicholas J. Sadgrove, Guillermo F. Padilla-González, Monique S. J. Simmonds, Igor Loncaric, Heidrun Kerschner, Petra Apfalter, Rainer Hartl, Ariane Deplano, Stien Vandendriessche, Barbora Černá Bolfíková, Pavel Hulva, Maiken C. Arendrup, Rasmus K. Hare, Céline Barnadas, Marc Stegger, Raphael N. Sieber, Robert L. Skov, Andreas Petersen, Øystein Angen, Sophie L. Rasmussen, Carmen Espinosa-Gongora, Frank M. Aarestrup, Laura J. Lindholm, Suvi M. Nykäsenoja, Frederic Laurent, Karsten Becker, Birgit Walther, Corinna Kehrenberg, Christiane Cuny, Franziska Layer, Guido Werner, Wolfgang Witte, Ivonne Stamm, Paolo Moroni, Hannah J. Jørgensen, Hermínia de Lencastre, Emilia Cercenado, Fernando García-Garrote, Stefan Börjesson, Sara Hæggman, Vincent Perreten, Christopher J. Teale, Andrew S. Waller, Bruno Pichon, Martin D. Curran, Matthew J. Ellington, John J. Welch, Sharon J. Peacock, David J. Seilly, Fiona J. E. Morgan, Julian Parkhill, Nazreen F. Hadjirin, Jodi A. Lindsay, Matthew T. G. Holden, Giles F. Edwards, Geoffrey Foster, Gavin K. Paterson, Xavier Didelot, Mark A. Holmes, Ewan M. Harrison, Anders R. Larsen

**Affiliations:** 1 Department of Bacteria, Parasites & Fungi, Statens Serum Institut, Copenhagen, Denmark; 2 Department of Veterinary Medicine, University of Cambridge, Cambridge, UK; 3 Royal Botanic Gardens, Kew, Richmond, UK; 4 Institute of Microbiology, University of Veterinary Medicine, Vienna, Austria; 5 National Reference Center for Antimicrobial Resistance and Nosocomial Infections, Institute for Hygiene, Microbiology and Tropical Medicine, Ordensklinikum Linz Elisabethinen, Linz, Austria; 6 National Reference Centre-Staphylococcus aureus, Department of Microbiology, Hôpital Erasme, Université libre de Bruxelles, Brussels, Belgium; 7 Department of Animal Science and Food Processing, Faculty of Tropical AgriSciences, Czech University of Life Sciences Prague, Prague, Czech Republic; 8 Department of Zoology, Charles University, Prague, Czech Republic; 9 Department of Biology and Ecology, University of Ostrava, Ostrava, Czech Republic; 10 European Programme for Public Health Microbiology Training (EUPHEM), European Centre for Disease Prevention and Control (ECDC), Stockholm, Sweden; 11 Infectious Disease Preparedness, Statens Serum Institut, Copenhagen, Denmark; 12 Department of Chemistry and Bioscience, Aalborg University, Aalborg, Denmark; 13 Wildlife Conservation Research Unit (WildCRU), Department of Zoology, University of Oxford, Tubney, UK; 14 Department of Veterinary and Animal Sciences, Faculty of Health and Medical Sciences, University of Copenhagen, Frederiksberg, Denmark; 15 National Food Institute, Technical University of Denmark, Kongens Lyngby, Denmark; 16 Expert Microbiology Unit, Department of Health Security, Finnish Institute for Health and Welfare, Helsinki, Finland; 17 Microbiology Unit, Finnish Food Authority, Helsinki, Finland; 18 Bacteriology Department and French National Reference Center for Staphylococci, Hospices Civils de Lyon, University of Lyon, Lyon, France; 19 Friedrich Loeffler-Institute of Medical Microbiology, University Medicine Greifswald, Greifswald, Germany; 20 Institute of Microbiology and Epizootics, Veterinary Faculty, Freie Universität Berlin, Berlin, Germany; 21 Institute for Veterinary Food Science, Justus-Liebig University Giessen, Giessen, Germany; 22 National Reference Centre for Staphylococci and Enterococci, Division Nosocomial Pathogens and Antibiotic Resistances, Department of Infectious Diseases, Robert Koch Institute, Wernigerode, Germany; 23 Vet Med Labor GmbH, Kornwestheim, Germany; 24 Dipartimento di Medicina Veterinaria, Università degli Studi di Milano, Lodi, Italy; 25 Norwegian Veterinary Institute, Ås, Norway; 26 Laboratory of Molecular Genetics, ITQB NOVA, Oeiras, Portugal; 27 Laboratory of Microbiology and Infectious Diseases, The Rockefeller University, New York, NY USA; 28 Servicio de Microbiología, Hospital Universitario Lucus Augusti, Lugo, Spain; 29 Department of Animal Health and Antimicrobial Strategies, National Veterinary Institute (SVA), Uppsala, Sweden; 30 Department of Microbiology, Public Health Agency of Sweden, Solna, Sweden; 31 Institute of Veterinary Bacteriology, University of Bern, Bern, Switzerland; 32 Department of Bacteriology, Animal and Plant Health Agency, Weybridge, UK; 33 Animal Health Trust, Newmarket, UK; 34 Antimicrobial Resistance and Healthcare Associated Infections Reference Unit, UK Health Security Agency, London, UK; 35 Clinical Microbiology and Public Health Laboratory, UK Health Security Agency, Addenbrooke’s Hospital, Cambridge, UK; 36 Department of Genetics, University of Cambridge, Cambridge, UK; 37 Department of Medicine, University of Cambridge, Cambridge, UK; 38 Institute of Infection and Immunity, St George’s, University of London, London, UK; 39 School of Medicine, University of St Andrews, St Andrews, UK; 40 Scottish MRSA Reference Laboratory, NHS Greater Glasgow and Clyde, Stobhill Hospital, Glasgow, UK; 41 SRUC Veterinary Services, Inverness, UK; 42 The Royal (Dick) School of Veterinary Studies and Roslin Institute, University of Edinburgh, Easter Bush, UK; 43 School of Life Sciences and Department of Statistics, University of Warwick, Warwick, UK; 44 Wellcome Sanger Institute, Hinxton, UK; 45 Department of Public Health and Primary Care, University of Cambridge, Cambridge, UK; 46 Present Address: Laboratory for Medical Microbiology, Ghent University Hospital, Ghent, Belgium; 47 Present Address: Advanced Light and Electron Microscopy (ZBS-4), Robert Koch Institute, Berlin, Germany; 48 Present Address: Quality Milk Production Services, Animal Health Diagnostic Center, Cornell University, Ithaca, NY, USA; 49 Present Address: Servicio de Microbiología, Complejo Asistencial Universitario de Salamanca, Salamanca, Spain; 50 Present Address: Department of Microbiology, Public Health Agency of Sweden, Solna, Sweden; 51 Present Address: Intervacc AB, Stockholm, Stockholm, Sweden; 52 Present Address: Department of Biomedical Science and Veterinary Public Health, Swedish University of Agricultural Sciences, Uppsala, Sweden; 53 Present Address: Antimicrobial Resistance and Healthcare Associated Infections Reference Unit, UK Health Security Agency, London, UK; 54 Present Address: Department of Physiology, Development & Neuroscience, University of Cambridge, Cambridge, UK

**Keywords:** Natural product synthesis, Population dynamics, Antimicrobial resistance, Bacterial evolution, Infectious-disease epidemiology

## Abstract

The discovery of antibiotics more than 80 years ago has led to considerable improvements in human and animal health. Although antibiotic resistance in environmental bacteria is ancient, resistance in human pathogens is thought to be a modern phenomenon that is driven by the clinical use of antibiotics^1^. Here we show that particular lineages of methicillin-resistant *Staphylococcus aureus*—a notorious human pathogen—appeared in European hedgehogs in the pre-antibiotic era. Subsequently, these lineages spread within the local hedgehog populations and between hedgehogs and secondary hosts, including livestock and humans. We also demonstrate that the hedgehog dermatophyte *Trichophyton erinacei* produces two β-lactam antibiotics that provide a natural selective environment in which methicillin-resistant *S. aureus* isolates have an advantage over susceptible isolates. Together, these results suggest that methicillin resistance emerged in the pre-antibiotic era as a co-evolutionary adaptation of *S. aureus* to the colonization of dermatophyte-infected hedgehogs. The evolution of clinically relevant antibiotic-resistance genes in wild animals and the connectivity of natural, agricultural and human ecosystems demonstrate that the use of a One Health approach is critical for our understanding and management of antibiotic resistance, which is one of the biggest threats to global health, food security and development.

## Abstract (web summary)

Methicillin-resistant strains of *Staphylococcus aureus* appeared in European hedgehogs in the pre-antibiotic era as a co-evolutionary adaptation to antibiotic-producing dermatophytes and have spread within the local hedgehog populations and between hedgehogs and secondary hosts.

## Main

Methicillin-resistant *S. aureus* (MRSA) is one of the most common antibiotic-resistant bacterial pathogens, causing approximately 171,000 invasive infections each year in Europe alone^2^. MRSA was first identified in 1960 shortly after the introduction of methicillin (celbenin) as a treatment option against penicillin-resistant *S*. *aureus* clones^3^, but was possibly selected for by the clinical use of penicillin over the previous 20 years^4^. Methicillin resistance has subsequently emerged in many *S*. *aureus* clones around the world, both in hospital and community settings as well as in livestock such as pigs and cattle^5,6^. This has serious implications for the treatment of severe infections and the World Health Organization now considers MRSA to be an important threat to human health^7^.

Methicillin resistance in *S*. *aureus* is mediated by the *mecA* and *mecC* genes, which encode the enzymes penicillin-binding protein 2a (PBP2a) and PBP2c, respectively. *mecA* and *mecC* confer resistance to almost all β-lactam antibiotics, including penicillinase-labile penicillins (such as penicillin G), penicillinase-stable penicillins (such as methicillin) and cephalosporins (such as cefoxitin).

Hedgehog surveys from Denmark and Sweden demonstrated a surprisingly high prevalence of MRSA carrying *mecC* (*mecC*-MRSA)^8,9^, raising the possibility that the evolution of these bacteria was driven by natural selection in wildlife, as opposed to clinical use of antibiotics. Historically, *mecC*-MRSA was first discovered in dairy cows and subsequently in humans^10^, suggesting that the use of antibiotics in livestock was providing a selective advantage and that human infections were the result of zoonotic transmission. Studies from many different European countries revealed that *mecC*-MRSA is also present in other domesticated animals such as sheep, goats and horses as well as in a broad range of wild animals, albeit at low frequencies^11^.

Our hypothesis that the evolution of *mecC*-MRSA was driven by natural selection is supported by studies from northwestern Europe and New Zealand that showed that hedgehogs are frequently colonized with the dermatophyte *T. erinacei*, which produces a penicillinase-labile penicillin-like substance that was recently identified as penicillin G^12–19^. To test our hypothesis, we examined the distribution of *mecC*-MRSA and other *S*. *aureus* isolates in hedgehogs in ten European countries and New Zealand. We sequenced 244 *S*. *aureus* isolates from hedgehogs and 913 *S*. *aureus* isolates from other sources to infer the evolutionary histories, host dynamics, geographical dispersal patterns and zoonotic potential of the major *mecC*-MRSA clones in Europe. The potential mechanisms for the natural selection of *mecC*-MRSA by *T*. *erinacei* were assessed by analysing the genome of *T*. *erinacei* for β-lactam biosynthetic genes and by screening *T*. *erinacei* for the production of β-lactams and antibiotic activity against a panel of *S*. *aureus* strains.

## The distribution of *mecC*-MRSA in hedgehogs

**Fig. 1:**
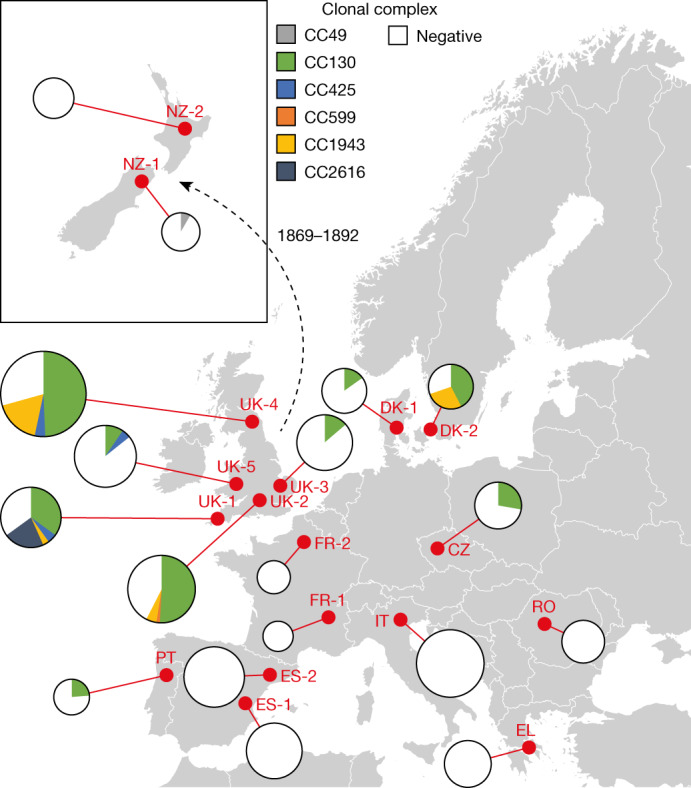
Distribution of *mecC*-MRSA clones in European and New Zealand hedgehog samples. The analysis included 828 samples from the nasal area, skin and feet of 276 hedgehogs originating from 16 wildlife rescue centres in 10 European countries and 2 wildlife rescue centres in New Zealand. The red dots indicate the sampling locations. The pie charts are connected to the sampling locations by a red line. The area of the pie chart is proportional to the number of samples from that location. The introduction of European hedgehogs into New Zealand from the UK between 1869 and 1892 is shown. A detailed description of the results is provided in Extended Data Fig. 1. Maps were provided by Eurostat under a Creative Commons Attribution 4.0 International (CC BY 4.0) licence; the administrative boundaries are copyright of EuroGeographics. Source data

**Extended Data Fig. 1:**
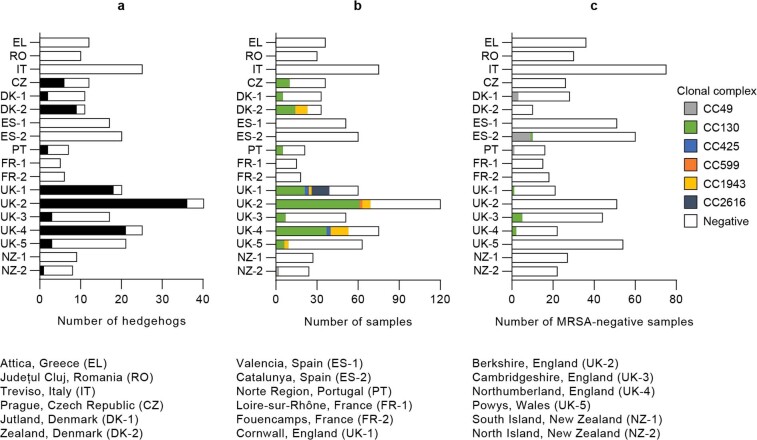
Distribution of *mecC*-MRSA clones in European and New Zealand hedgehogs. The analysis included 828 samples from the nasal area, skin and feet of 276 hedgehogs originating from 16 wildlife rescue centres in ten European countries and two wildlife rescue centres in New Zealand. **a**, Presence of *mecC*-MRSA in hedgehogs (n = 276). Presence and absence are shown as black and white boxes, respectively. **b**, Distribution of *mecC*-MRSA clones in hedgehog samples (n = 828). **c**, Distribution of MSSA clones in MRSA-negative hedgehog samples (n = 606). Source data

We first examined the geographical distribution and population structure of *mecC*-MRSA in European hedgehogs, which inhabit large parts of Europe as a result of postglacial expansion from Pleistocene refugia^20^. European hedgehogs have also become widespread in New Zealand after a series of introductions from the UK between 1869 and 1892 (ref. ^21^). We analysed 828 samples from the nasal area, skin and feet of 276 hedgehogs originating from 16 wildlife rescue centres in 10 European countries and 2 wildlife rescue centres in New Zealand (Fig. 1 and Extended Data Fig. 1). *mecC*-MRSA was present in 101 of the 172 hedgehogs (222 out of 516 samples) from England and Wales (66%, 81 out of 123), Czech Republic (50%, 6 out of 12), Denmark (50%, 11 out of 22), Portugal (29%, 2 out of 7) and New Zealand (6%, 1 out of 17), therefore extending the known geographical distribution of *mecC*-MRSA in hedgehogs (Fig. 1 and Extended Data Fig. 1). By contrast, all 104 hedgehogs (312 samples) from Greece, Romania, Italy, France and Spain tested negative for *mecC*-MRSA. Whole-genome sequencing showed that the 222 *mecC*-MRSA isolates belonged to 6 clonal complexes, CC130 (75%), CC1943 (15%), CC2616 (6%), CC425 (3%), CC49 (1%) and CC599 (1%), of which CC130 had the most widespread distribution across western and central Europe (Fig. 1 and Extended Data Fig. 1). We screened all of the MRSA-negative hedgehog samples from our study (*n* = 606) for the presence of methicillin-susceptible *S*. *aureus* (MSSA) isolates belonging to the same clonal complexes as the *mecC*-MRSA isolates (Extended Data Fig. 1). This led to the identification of 22 MSSA isolates, including 13 CC49 isolates from Spain (*n* = 9), Denmark (*n* = 3) and Portugal (*n* = 1), and 9 CC130 isolates from England (*n* = 8) and Spain (*n* = 1).

The *mecC* gene encoding PBP2c is located immediately upstream of a *blaZ* gene (hereafter, *blaZ*_LGA251_) on a chromosomally integrated mobile genetic element known as a type XI staphylococcal cassette chromosome *mec* (SCC*mec*). PBP2c and the *blaZ*_LGA251_-encoded penicillinase are orthologues of the PBP2a enzyme and penicillinase produced by other *S*. *aureus* clones, although they share only 63% and 65% amino acid identities with each other, respectively^10^. Penicillinases have a narrower spectrum than PBP2a and PBP2c and provide resistance only to penicillin G and other penicillinase-labile subclasses of penicillin. As expected, *blaZ*_LGA251_ was present in the 222 *mecC*-MRSA isolates but absent in the 22 MSSA isolates. However, 14 of the MSSA isolates carried the *blaZ* gene found in other *S*. *aureus* clones (Supplementary Table 1).

## Production of β-lactams by *T*. *erinacei*

**Fig. 2:**
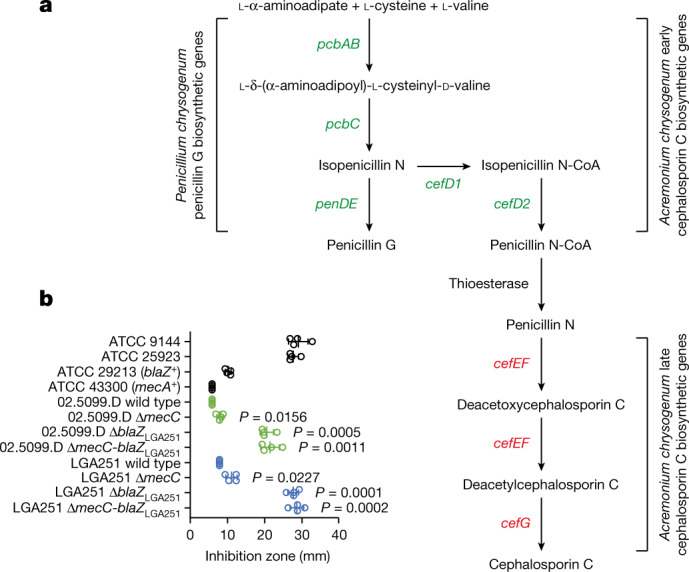
Penicillin biosynthetic genes and antibiotic activity of *T*. *erinacei* IMI 101051. **a**, Schematic of the key steps in the biosynthesis of penicillin G and cephalosporin C. The presence (green) or absence (red) of *T*. *erinacei* penicillin G and cephalosporin C biosynthetic genes is indicated. **b**, *T.* *erinacei* inhibition zones against a collection of *S*. *aureus* control strains (black) and two *mecC*-MRSA wild-type strains belonging to CC130 (green) and CC425 (blue) and their isogenic mutants. Two-tailed paired Student’s *t*-tests were used to compare inhibition zones of each mutant to the corresponding wild-type strain. Data are mean ± s.d.; *n* = 4 biologically independent fungal culture extracts. A detailed description of the results is provided in Extended Data Fig. 4. Source data

The abundance of *mecC*-MRSA in hedgehogs led us to speculate that antibiotic production by *T*. *erinacei* provides a selective environment in which *mecC*-MRSA isolates have an advantage over susceptible isolates. Genome sequencing and analysis of the *T*. *erinacei* type strain IMI 101051 (ATCC 28443) identified orthologues of *pcbAB*, *pcbC* and *penDE*, which are responsible for key steps in penicillin G production by *Penicillium chrysogenum*, as well as the *Acremonium chrysogenum* early cephalosporin C biosynthetic genes *cefD1* and *cefD2*, which are involved in the conversion of isopenicillin N into penicillin N (Fig. 2 and Extended Data Table 1). By contrast, *T*. *erinacei* IMI 101051 lacked the *A*. *chrysogenum* late cephalosporin C biosynthetic genes *cefEF* and *cefG*. *P*. *chrysogenum* also carries *cefD1* and *cefD2* but is nevertheless incapable of producing cephalosporins due to the lack of *cefEF* and *cefG*^22,23^.

**Extended Data Fig. 2:**
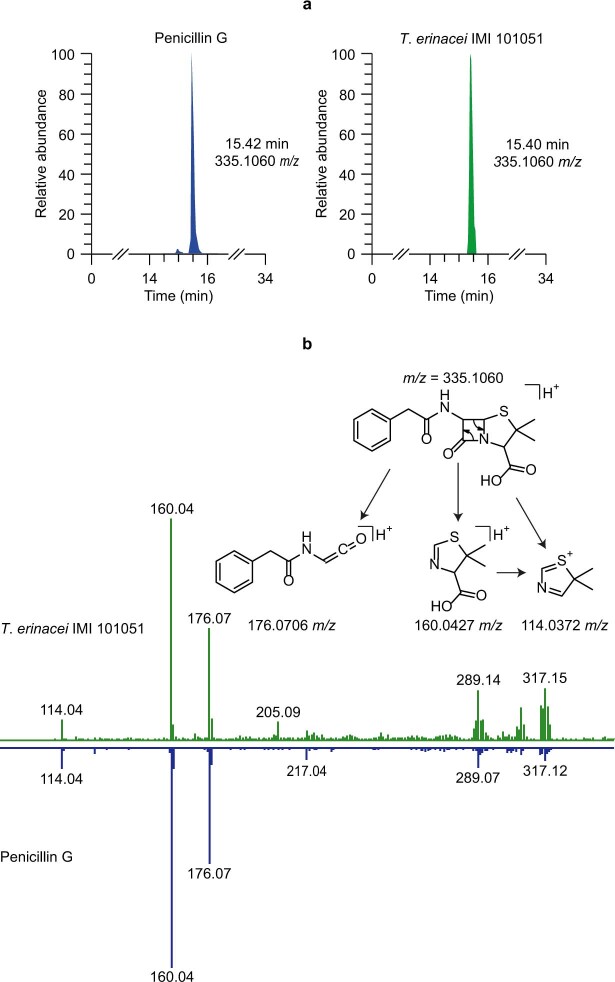
Detection of penicillin G in *T*. *erinacei* IMI 101051 culture broth. **a**, Left and right panels show extracted ion chromatogram of a pure standard of penicillin G and in *T*. *erinacei* culture broth, respectively. **b**, Upper and lower panels show MS^2^ spectra of penicillin G in a *T*. *erinacei* culture broth and a pure standard of penicillin G, respectively.

**Extended Data Fig. 3:**
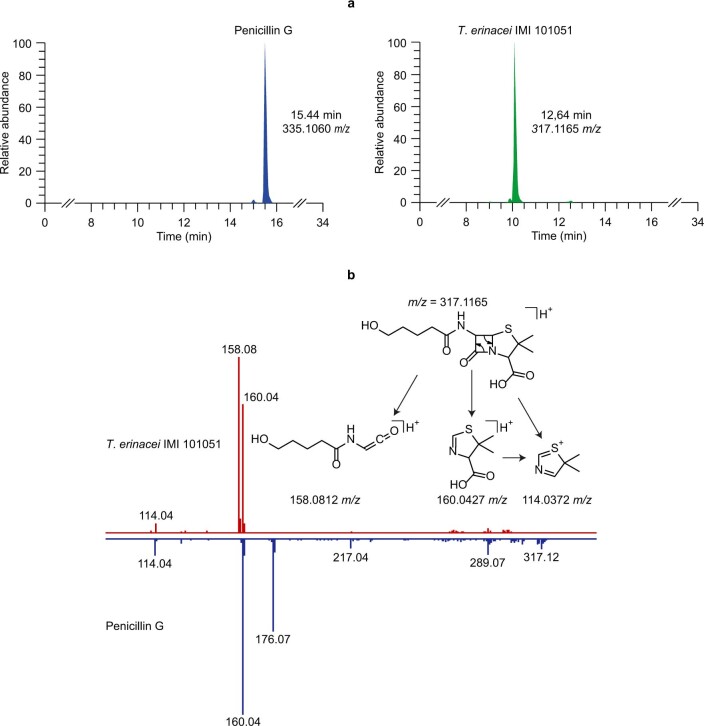
Detection of KPN in *T*. *erinacei* IMI 101051 culture broth. **a**, Left and right panels show extracted ion chromatogram of a pure standard of penicillin G and KPN in *T*. *erinacei* culture broth, respectively. **b**, Upper and lower panels show MS^2^ spectra of KPN in a *T*. *erinacei* culture broth and a pure standard of penicillin G, respectively.

We processed four distinct culture broths of *T*. *erinacei* IMI 101051 for metabolic profiling using liquid chromatography–mass spectrometry (LC–MS) and molecular networking analysis. This led to the identification of two β-lactams, penicillin G and 6-(5-hydroxy-*n*-valeramido)-penicillanic acid (KPN), both of which belong to the penicillin class of antibiotics (Extended Data Figs. 2 and 3 and Supplementary Fig. 1). KPN has to date been found only in culture broths of fungal strains belonging to the genus *Paecilomyces*^24^ and differs from penicillin G by having a unique side chain (Extended Data Figs. 2 and 3 and Supplementary Fig. 1). The biosynthetic pathway of KPN is currently unknown.

## Selection of *mecC*-MRSA by *T*. *erinacei*

**Extended Data Fig. 4:**
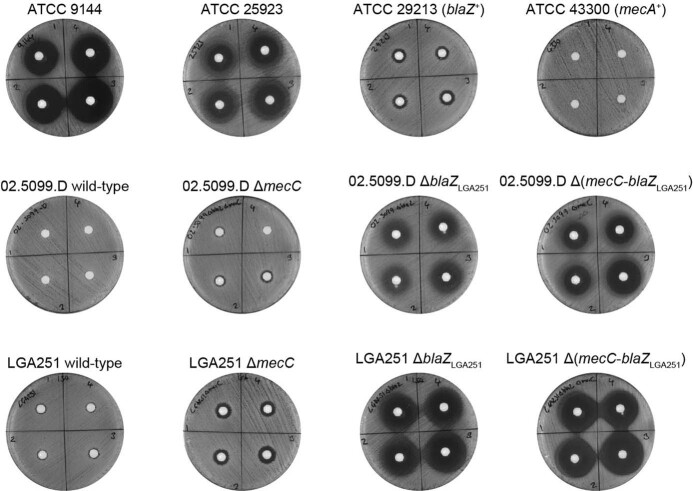
Antibiotic activity of *T*. *erinacei* IMI 101051. *T*. *erinacei* inhibition zones against a collection of *S*. *aureus* control strains and two *mecC*-MRSA wild-type strains belonging to CC130 (02.5099.D) and CC425 (LGA251) and their isogenic mutants. The numbers on the plates refer to each of four biologically independent fungal culture extracts. Source data

Four culture broths of *T*. *erinacei* IMI 101051 were screened for antibiotic activity against a collection of *S*. *aureus* control strains. All of the culture broths produced large inhibition zones against two penicillin-susceptible *S*. *aureus* strains—ATCC 9144 (Oxford *S*. *aureus*) and ATCC 25923—but much smaller zones against the penicillinase-producing *S*. *aureus* strain ATCC 29213 and the *mecA*-positive *S*. *aureus* strain ATCC 43300 (Fig. 2 and Extended Data Fig. 4). The role of *mecC* and *blaZ*_LGA251_ was assessed by screening the culture broths for antibiotic activity against two *mecC*-MRSA wild-type strains belonging to CC130 (02.5099.D) and CC425 (LGA251) and their isogenic mutants. The mutants with deleted *mecC* (Δ*mecC*)*, blaZ*_LGA251_ (Δ*blaZ*_LGA251_), and *mecC* and *blaZ*_LGA251_ (Δ*mecC*-*blaZ*_LGA251_) produced significantly larger inhibition zones compared with the corresponding wild-type strains, although the zones of the Δ*blaZ*_LGA251_ and Δ*mecC*-*blaZ*_LGA251_ mutants were larger compared with the zones of the Δ*mecC* mutants (Fig. 2 and Extended Data Fig. 4). These results indicate that *mecC* and *blaZ*_LGA251_ both contribute to the reduced susceptibility of *mecC*-MRSA to penicillin G and KPN present in culture broths of *T*. *erinacei* IMI 101051.

## Evolutionary history of *mecC*-MRSA

We sought to infer the evolutionary histories of *S*. *aureus* CC130, CC425 and CC1943, which constitute the most successful *mecC*-MRSA clones in Europe^10,11,25^. For this purpose, we collected and sequenced 786 *mecC*-MRSA and 127 MSSA CC130, CC425 and CC1943 isolates selected to represent the known geographical distribution (mainly western and central Europe) and host repertoire (mainly humans, cattle, sheep, goats and wild animals) of each clone (Supplementary Table 1). We used core-genome single-nucleotide polymorphism (SNP) diversity and isolation dates to infer time-scaled phylogenies of these isolates and the 205 *mecC*-MRSA and 9 MSSA CC130, CC425 and CC1943 isolates collected from hedgehogs (Supplementary Table 1). The sequencing data were processed for pan-genome analysis to identify antibiotic-resistance genes (ARGs) and mobile genetic elements that encode human- and ruminant-specific immune modulators that are involved in host switching events, including a phage-encoded immune evasion cluster-1 (IEC-1) enabling *S*. *aureus* to evade the human innate immune response and a staphylococcal pathogenicity island (SaPI)-encoded *vwb* gene (*vwb*_SaPI_), which encodes a von Willebrand factor-binding protein with coagulase activity against ruminant plasma^26^.

**Fig. 3:**
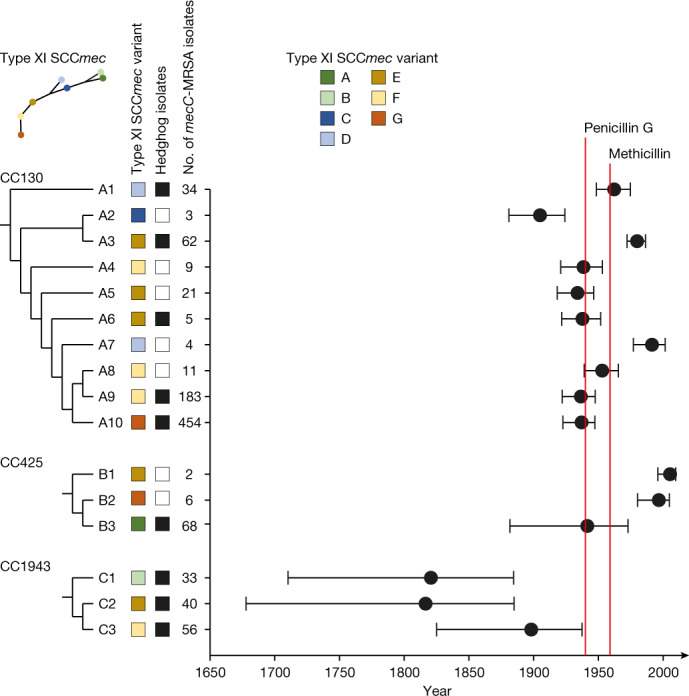
Timeline of *mecC*-MRSA CC130, CC425 and CC1943 evolution in Europe. Manual mapping of the tips on the type XI SCC*mec* phylogeny onto the CC130, CC425 and CC1943 phylogenies, and vice versa, enabled us to assign the *mecC*-MRSA isolates to 16 monophyletic lineages containing orthologous type XI SCC*mec* elements (A–G). The trees are redrawn from Supplementary Figs. 2–5 to illustrate the branching order of the different type XI SCC*mec* variants and *mecC*-MRSA lineages. Branch lengths are not drawn to scale. The presence and absence of hedgehog isolates in a given lineage are shown as black and white boxes, respectively. A detailed description of the geographical distribution and host range of major *mecC*-MRSA CC130, CC425 and CC1943 lineages is provided in Extended Data Fig. 7. The estimated date of the most recent common ancestor and 95% confidence interval of each *mecC*-MRSA lineage are illustrated by filled circles and horizontal lines, respectively. The introduction of penicillin G and methicillin as therapeutic options is indicated by red lines. Source data

**Extended Data Fig. 5:**
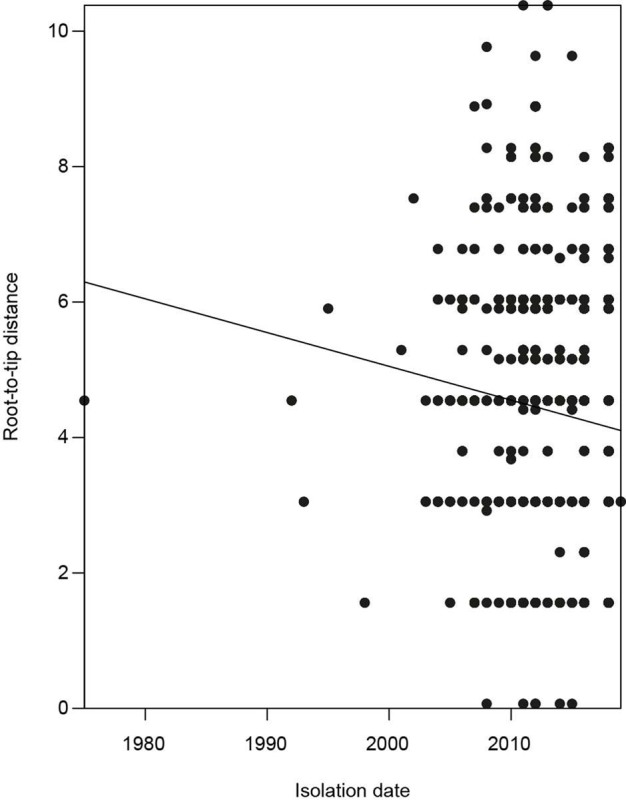
Root-to-tip linear regression analysis of the type XI SCC*mec* dataset. The correlation between root-to-tip distances and isolation dates is very weak with a coefficient of determination *R*^2^ = −0.05.

We also sought to infer a time-scaled phylogeny of the 991 type XI SCC*mec* elements containing the *mecC* and *blaZ*_LGA251_ genes but the correlation between root-to-tip distances and isolation dates was too weak with a coefficient of determination *R*^2^ = −0.05 (Extended Data Fig. 5). Instead, we used the topology of the type XI SCC*mec* phylogeny to identify monophyletic *mecC*-MRSA lineages harbouring orthologous type XI SCC*mec* elements. The type XI SCC*mec* elements could be traced back to seven nodes that were connected to each other on a long backbone. Each of the backbone nodes and its orthologous descendants received the same letter designation to reflect their genetic relationship (A to G) (Fig. 3 and Supplementary Fig. 2). Manual mapping of the tips onto the CC130, CC425 and CC1943 phylogenies, and vice versa, enabled us to assign the *mecC*-MRSA isolates to 16 monophyletic lineages harbouring orthologous type XI SCC*mec* elements (Fig. 3 and Supplementary Figs. 2–5). The 129 *mecC*-MRSA CC1943 isolates could be divided into three lineages (C1 to C3), which probably originated in the early-to-late 1800s, long before the first β-lactam—penicillin G—became widely available as a therapeutic option in the 1940s (Fig. 3). The 786 *mecC*-MRSA CC130 isolates and 76 *mecC*-MRSA CC425 isolates belonged to 10 and 3 lineages (A1 to A10 and B1 to B3, respectively) (Fig. 3). Several of these lineages also originated in the pre-antibiotic era (Fig. 3). Most *mecC*-MRSA isolates lacked *vwb*_SaPI_ (96%, 949 out of 991) and IEC-1 (100%, 990 out of 991) and were genotypically susceptible to non-β-lactam antibiotics (Supplementary Figs. 3–5). The largest *mecC*-MRSA CC425 lineage (CC425:B3) had a unique evolutionary trajectory with signs of adaptation to ruminants (Supplementary Fig. 4). The basal *mecC*-MRSA CC425:B3 isolates probably originated in England during the early 1940s and shared epidemiological and genetic characteristics with the other *mecC*-MRSA lineages: they were associated with multiple hosts, including hedgehogs, cattle and humans, and lacked *vwb*_SaPI_ and IEC-1. By contrast, their descendants (CC425:B3.1) harboured *vwb*_SaPI_, were restricted to cattle and humans in southwest England and probably diverged during the 1960s (date of the most recent common ancestor (MRCA), 1965; 95% confidence interval (CI), 1926–1986). Our analysis revealed that some of the *mecC*-MRSA lineages (CC130:A2, CC425:B3 and CC1943:C1) carried unique variants of the type XI SCC*mec* element, supporting that they have evolved through vertical inheritance from the MRCA of each *mecC*-MRSA lineage, whereas others shared the same type XI SCC*mec* variant (Fig. 3 and Supplementary Figs. 2–5). The latter findings could be the result of either purifying (negative) selection, convergent evolution, homologous recombination between different *mecC*-MRSA lineages or horizontal gene transfer.

**Extended Data Fig. 6:**
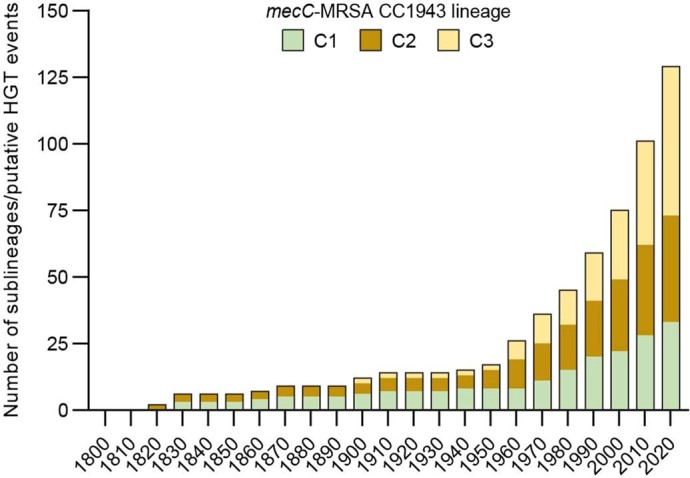
Number of *mecC*-MRSA CC1943 sublineages. The bars show the number of sublineages of *mecC*-MRSA CC1943:C1, *mecC*-MRSA CC1943:C2 and *mecC*-MRSA CC1943:C3 at different time points. Source data

**Extended Data Fig. 7:**
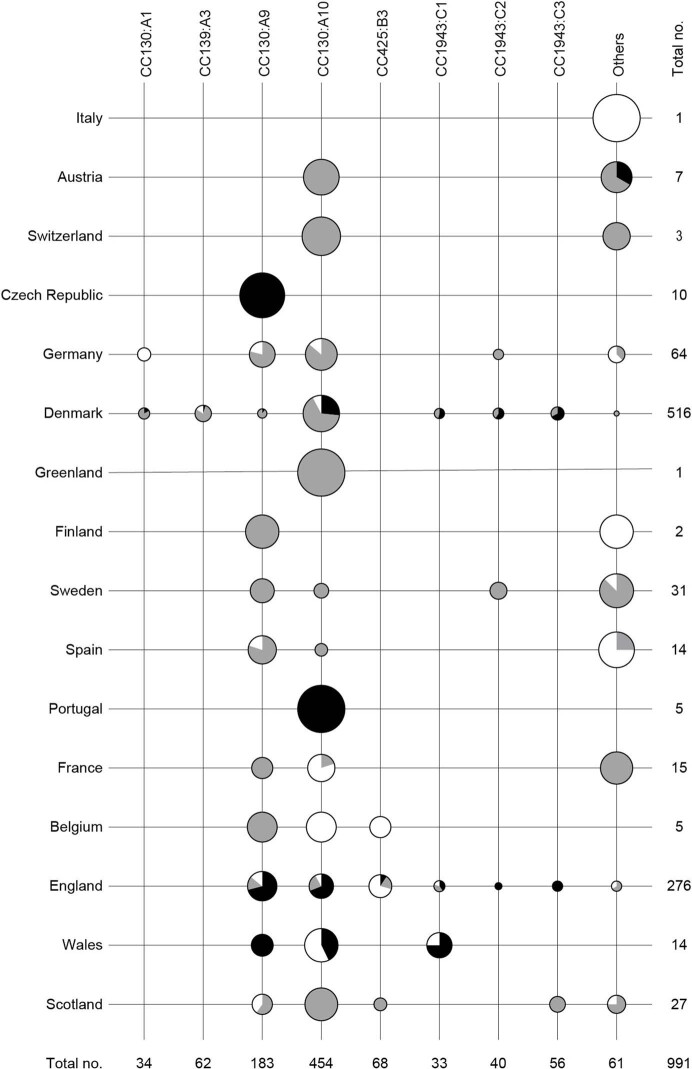
Geographical distribution and host range of major *mecC*-MRSA CC130, CC425 and CC1943 lineages. The study collection included 991 *mecC*-MRSA CC130, CC425 and CC1943 isolates originating from 16 European countries. The countries of sampling and the eight largest (≥25 isolates) *mecC*-MRSA lineages are shown. Isolates belonging to the eight minor *mecC*-MRSA lineages are grouped together (others). The pie charts depict the proportion of *mecC*-MRSA isolates from hedgehogs (black), humans (grey) and other sources (white). The area of the pie chart is proportional to the number of *mecC*-MRSA isolates from that country. Source data

To better understand the potential role of horizontal gene transfer in the evolution of the three early *mecC*-MRSA CC1943 lineages, we determined the smallest number of sublineages that were present at a given time point, which is also the smallest number of acquisition events that could explain the presence of the same type XI SCC*mec* variant in all sublineages (Extended Data Fig. 6). The analysis showed that *mecC*-MRSA CC1943 consisted of 15 sublineages in 1940, just before penicillin G became available as a therapeutic option. It has previously been estimated that SCC*mec* was acquired in a single horizontal gene transfer event in three of the major hospital- and community-associated MRSA clones^4,27,28^, although multiple introductions have also been reported^29^. Thus, it is more plausible to assume that the type XI SCC*mec* element was present in the MRCA of each *mecC*-MRSA CC1943 lineage rather than assuming horizontal gene transfer into each of the 15 sublineages within a few years at the beginning of the antibiotic era. Vertical inheritance of the type XI SCC*mec* element is also consistent with the apparent absence of admixture between the different *mecC*-MRSA CC1943 lineages despite the fact that they are often found in hedgehogs within the same geographical area (Extended Data Fig. 7). Notably, the type XI SCC*mec* variants found in *mecC*-MRSA CC1943:C2 (E) and CC1943:C3 (F) were each other’s neighbours on the type XI SCC*mec* phylogeny, and it is therefore possible that the type XI SCC*mec* element was acquired even earlier (date of the MRCA, 1737; 95% CI, 1562–1824).

MSSA isolates comprised 8% (67 out of 851), 47% (68 out of 144) and 0.8% (1 out of 130) of the CC130, CC425 and CC1943 isolates, respectively. The vast majority of the MSSA CC130 and MSSA CC425 isolates, but not the single MSSA CC1943 isolate, were basal to the corresponding *mecC*-MRSA lineages (Supplementary Figs. 3–5). Most of the basal MSSA CC130 isolates originated from sheep and goats in Italy, France, Spain and Norway and harboured *vwb*_SaPI_. Moreover, some of the isolates carried ARGs against antibiotics that are used to treat infections in sheep and goats, including the two tetracycline-resistance genes *tet*(K) and *tet*(L), the *blaZ* gene found in other *S*. *aureus* clones, the chloramphenicol resistance gene *cat* and the macrolide resistance gene *erm*(C). The earliest branching CC425 lineages were epidemiologically and genetically diverse with respect to host range and the presence/absence of the type XI SCC*mec* element, *vwb*_SaPI_ and ARGs, although most originated from wild animals in Spain and lacked the aforementioned genetic determinants. Together, these findings suggest that CC130 and CC425 emerged from distinct ruminant and wildlife reservoirs in Europe and that methicillin resistance is an acquired phenotype within these clones.

## Population dynamics of *mecC*-MRSA

Hedgehogs constitute a large reservoir of *mecC*-MRSA clones, as demonstrated here and elsewhere^8,9^, whereas *mecC*-MRSA isolates are present at much lower frequencies in humans, domesticated animals and other wild animals. Hedgehog isolates were present in 9 out of the 16 *mecC*-MRSA lineages, including the 8 largest (≥25 isolates) and 3 earliest (200–130 years ago) lineages (Fig. 3). The 2 largest *mecC*-MRSA CC130 lineages (CC130:A9 and CC130:A10) encompassed 67% (232 out of 344), 65% (339 out of 520) and 43% (66 out of 153) of all *mecC*-MRSA isolates from hedgehogs, humans and other sources, respectively, and had the broadest geographical ranges in western and central Europe (Extended Data Fig. 7 and Supplementary Figs. 3–5). Several of the major *mecC*-MRSA CC130 and CC1943 lineages (such as CC130:A9, CC130:A10, CC1943:C1, CC1943:C2 and CC1943:C3) contained isolates that were separated by wide expanses of seawater, reflecting numerous long-distance dispersal events between British and Danish islands and mainland Europe within the past 200 years (Extended Data Fig. 7 and Supplementary Figs. 3–5). By contrast, analysis of the fine-scale population structure of *mecC*-MRSA isolates from hedgehogs revealed a detailed pattern of diversifications over short distances, resulting in substantial concordance between genetic clusters and geography at the local level (Supplementary Figs. 3–5). The observed clustering is consistent with the limited dispersal capacity of hedgehogs and the effects of habitat fragmentation.

**Fig. 4:**
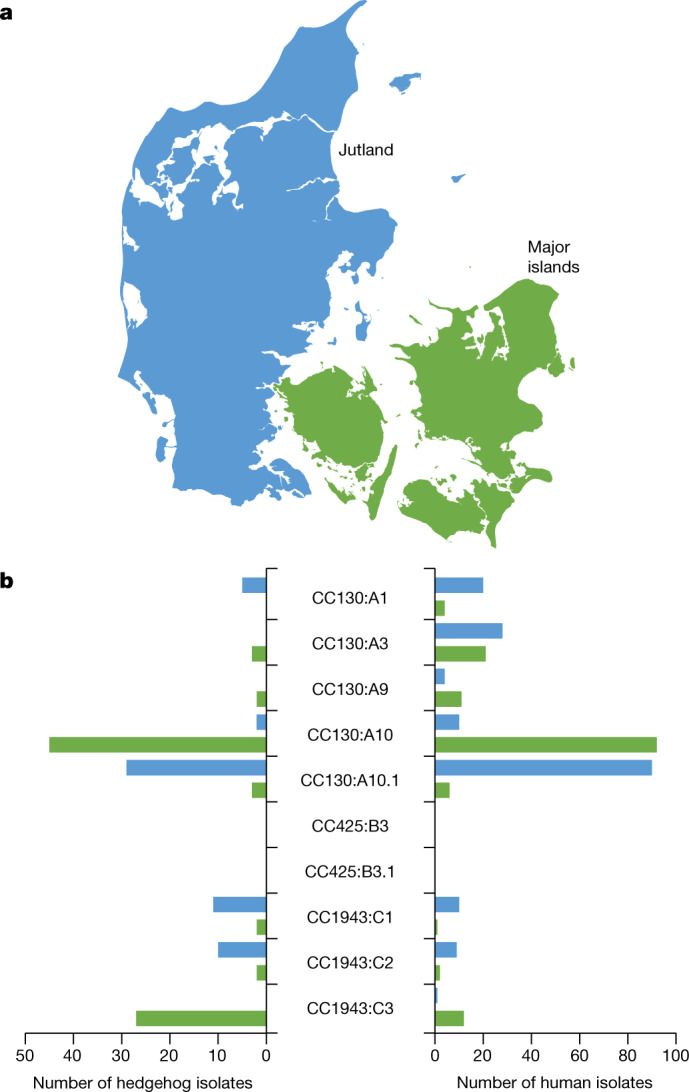
Population structures of Danish *mecC*-MRSA isolates from hedgehogs and humans. **a**, The map of Denmark shows the geographical ranges of two of the three hedgehog subpopulations in Jutland and on the major islands. **b**, The geographical distribution of major *mecC*-MRSA CC130, CC425 and CC1943 lineages in two broad collections of *mecC*-MRSA isolates recovered from hedgehogs (*n* = 141) and humans (*n* = 327) in Jutland and on the major islands. Hedgehog and human isolates from the remaining hedgehog subpopulation on the small island of Bornholm (not shown) were excluded from the analysis due to their small sample size (*n* = 9). A detailed map of the sampling locations is provided in Extended Data Fig. 8. Maps were provided by Eurostat under a Creative Commons Attribution 4.0 International (CC BY 4.0) licence; the administrative boundaries are copyright of EuroGeographics. Source data

**Extended Data Fig. 8:**
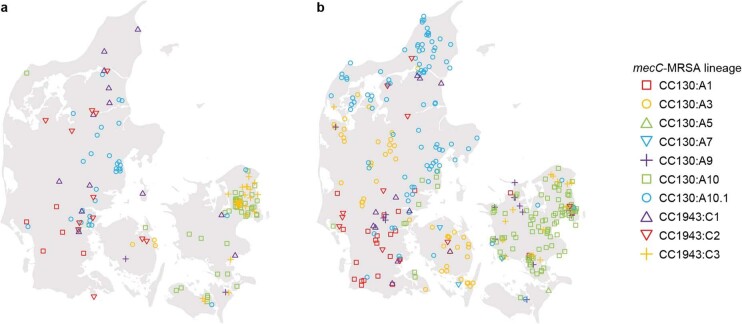
Sampling locations of Danish *mecC*-MRSA isolates from hedgehogs and humans. The maps of Denmark relate to the map shown in Fig. 4. **a**, *mecC*-MRSA recovered from hedgehogs (n = 141). **b**, *mecC*-MRSA recovered from humans (n = 327). The location of each sample is given at the zip code area level. Maps were provided by Eurostat under a Creative Commons Attribution 4.0 International (CC BY 4.0) licence; the administrative boundaries are copyright of EuroGeographics.

Assuming that hedgehogs act as local reservoirs (sources) of *mecC*-MRSA in Denmark, theoretical considerations predict that secondary transmission to other hosts (sinks) would lead to similar patterns of genetic variation within these secondary hosts cohabiting the same geographical region. To examine this theory, we determined the local population structures of two broad collections of Danish *mecC*-MRSA isolates from hedgehogs and humans covering the geographical ranges of two of the three Danish hedgehog subpopulations, namely Jutland (a peninsula connected to continental Europe) and major islands^30^. Hedgehog and human isolates from the geographical range of the remaining hedgehog subpopulation (the small island of Bornholm) were excluded from the analysis due to their small sample size (nine isolates). Most of the CC130:A10 isolates from Jutland formed a distinct sublineage (CC130:A10.1) together with isolates from other parts of Europe (Supplementary Fig. 3). As a consequence, CC130:A10.1 isolates were treated as a separate group in the analysis. The results revealed distinct patterns of regional dispersal with little overlap between Jutland and the major islands and a notable correlation between the population structures of hedgehog and human isolates at the regional level (*P* = 0.0149, two-sided Wilcoxon matched-pairs signed-rank test) (Fig. 4 and Extended Data Fig. 8). These findings support the hypothesis that human isolates originate from local hedgehog reservoirs, although it should be noted that the data presented here do not provide evidence for directionality or rule out the involvement of other animal reservoirs (such as livestock) as part of the transmission chains.

## Discussion

This research shows that hedgehogs are a natural reservoir of zoonotic *mecC*-MRSA lineages that predate the antibiotic era, which is inconsistent with the commonly accepted view that widespread resistance in clinical pathogens is a modern phenomenon that is driven by our use of antibiotics in human and veterinary medicine.

Data on the prevalence of *mecC*-MRSA in humans and different animal species indicate that hedgehogs are the most likely primary host in some countries. For example, in Denmark, the prevalence in hedgehogs is considerably higher than in cattle (veal calves and bulk tank milk), sheep and goats (61% versus 0.0–1.1%)^8,31,32^, and the number of human cases is relatively low (3–36 cases per year)^25^. This is further supported by our finding that *mecC*-MRSA generally lacks genetic markers of human and ruminant adaptation, with the notable exception of the CC425:B3.1 lineage that has undergone a host jump from hedgehogs to cattle in southwest England. Before this study, dairy cows were considered to be the most likely reservoir of *mecC*-MRSA and a major source of zoonotic infections in humans. This hypothesis was supported by the fact that β-lactams are commonly used to treat bovine mastitis, as shown by sales data from Denmark and the UK^33,34^. However, our findings strongly suggest that most *mecC*-MRSA lineages originate from hedgehogs, although dairy cows and other domesticated animals probably act as intermediate hosts and vectors in zoonotic transmission from hedgehogs to humans, as previously demonstrated^35^.

Here we show that the *T*. *erinacei* type strain IMI 101051 from New Zealand produces two β-lactams—penicillin G and KPN—and that *mecC* and *blaZ*_LGA251_ both contribute to reduced susceptibility of *mecC*-MRSA to these antibiotics. Previous studies have established that *T*. *erinacei* is widespread among hedgehogs in New Zealand and northwestern Europe and that isolates from both continents produce a penicillinase-labile penicillin-like substance^12–19^, and a recent study characterized penicillin G from *T*. *erinacei* in Sweden (the presence of other β-lactams was not investigated)^19^. This suggests that penicillin-producing *T*. *erinacei* isolates were circulating in European hedgehogs long before they were introduced into New Zealand in the late 1800s and that methicillin resistance first emerged in Europe as a co-evolutionary adaptation of *S*. *aureus* to colonization of hedgehogs. By contrast, it cannot be ruled out that clinical use of antibiotics in humans and livestock has contributed to the evolution of some of the younger *mecC*-MRSA CC130 and CC425 lineages, although only one of these lineages (CC425:B3) showed signs of adaptation to either of these hosts. Our findings indicate that seven of the younger *mecC*-MRSA lineages (CC130:A3, CC130:A4, CC130:A5, CC130:A6, CC130:A8, CC130:A9 and CC425:B1) have acquired their type XI SCC*mec* variants from the early *mecC*-MRSA CC1943:C2 and CC1943:C3 lineages (Fig. 3 and Supplementary Figs. 2–5). The type XI SCC*mec* element has also been found at low frequencies in coagulase-negative *Staphylococcus* species from wild animals and livestock but the evolutionary links between these potential donors and *mecC*-MRSA remain to be investigated^36^.

Our analyses suggest that most *mecC*-MRSA transmission events within hedgehog populations and between hedgehogs and secondary hosts are highly localized. The finding that some human *mecC*-MRSA isolates probably originate from local hedgehog reservoirs indicates that *mecC*-MRSA has been a cause of sporadic infections in humans for the past 200 years, more than a century before MRSA was first identified in patients in 1960 (ref. ^3^). The host interactions that lead to zoonotic transmission probably include direct contact with hedgehogs or contact with secondary animal hosts such as dairy cows, as previously shown for *T*. *erinacei* (the cause of ‘hedgehog ringworm’ in humans)^13^. We also identified several long-distance dispersal events between British and Danish islands and mainland Europe. The connections that bridge geographically isolated hedgehog populations are poorly understood but might involve oversea movements of humans and livestock. Furthermore, a recent report of *mecC*-MRSA in white storks raises the possibility that migratory birds could be efficient long-distance carriers^37^.

β-Lactams target PBPs that catalyse carboxypeptidase and transpeptidase reactions during bacterial cell wall synthesis, whereby they inhibit cross-linking of neighbouring peptidoglycan strands. The primary mechanisms of β-lactam resistance in *S*. *aureus* are enzymatic cleavage of the amide bond in the β-lactam ring of penicillinase-labile penicillins by *blaZ*-encoded penicillinases and the production of *mecA*- or *mecC*-encoded PBP2a and PBP2c, respectively, with a decreased affinity for a broad spectrum of β-lactams. Our findings support that both mechanisms contribute to protection against penicillin production by *T*. *erinacei* in hedgehogs, although it should be noted that the Δ*mecC* mutants produced smaller inhibition zones than the Δ*blaZ*_LGA251_ mutants. This might be due to the fact that PBP2c has a relatively high binding affinity for penicillins compared with PBP2a^38^. It is also possible that PBP2c provides additional ecological benefits, such as protection against cephalosporin-producing fungi and bacteria that occur naturally in all environments^39^.

*mecC* and *blaZ*_LGA251_ have also been found on a pseudo-SCC*mec* element (ΨSCC*mec*_P5085_) in *Staphylococcus edaphicus*, a soil-dwelling bacterial species isolated from Antarctica^40^. In contrast to the type XI SCC*mec* element, ΨSCC*mec*_P5085_ lacks the cassette chromosome recombinase (*ccr*) genes that are responsible for movement (excision and integration) of SCC*mec*^40^. Several studies have identified ARGs in ancient and modern samples and bacteria from natural environment such as soil^41–43^. Yet, the environmental resistome shows limited potential for horizontal gene transfer and, for this reason, the contribution of environmental ARGs to resistance in human pathogens has so far been controversial^44^. It seems reasonable to assume that the microbiota of wild animals have greater exposure to the environmental resistome than the human microbiota and are therefore more likely to acquire environmental ARGs. Thus, wild animals might represent a hitherto unrecognized conduit through which environmental ARGs can be transferred to clinical pathogens.

**Extended Data Fig. 9:**
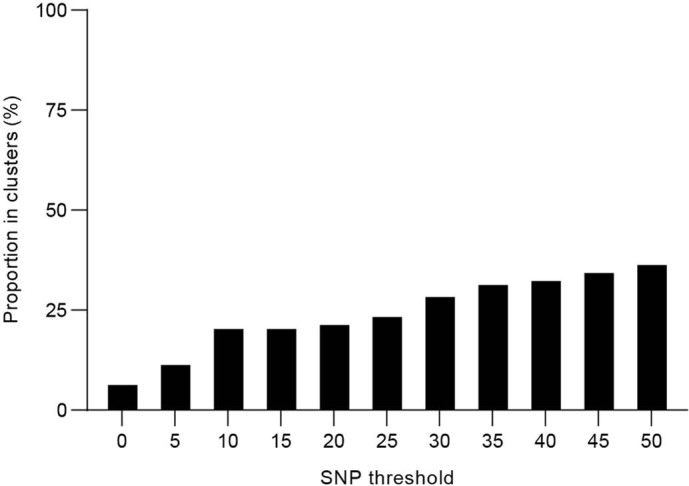
Frequency of potential transmission events of *mecC*-MRSA CC130 isolates within wildlife rescue centres. The bars show the proportion of isolates that belong to clusters defined at different pairwise SNP distance thresholds. Source data

We acknowledge some limitations of our study. Although we broadly sampled hedgehogs across Europe and New Zealand, our study represents only a small part of the geographical range and a small number of hedgehog samples in most countries. Thus, the distribution and diversity of *mecC*-MRSA in Europe and New Zealand might be larger than documented here. We cannot account for the potential effect of transmission within the different wildlife rescue centres. To better understand the transmission dynamics, we examined the frequency of potential transmission events of *mecC*-MRSA CC130 within the different facilities using a range of maximum pairwise SNP distance thresholds to define a cluster (Extended Data Fig. 9). Using a conservative cut-off of 25 SNPs (transmission age < 6 months)^45^, 25% (683 out of 2,783) of the *mecC*-MRSA CC130 isolate pairs collected within the same facility belonged to a potential transmission cluster. It is therefore possible that the prevalence of *mecC*-MRSA in hedgehogs kept in such facilities is higher than in the wild, although it is also probable that some of these potential transmission events occurred in the wild before admission to the facility. Notably, a previous study found that there is only a slightly lower prevalence of *mecC*-MRSA in hedgehogs that died in the wild compared with hedgehogs that died while staying in a wildlife rescue centre^8^, which is consistent with our finding that most hedgehogs acquired *mecC*-MRSA outside the facility. We were unable to test the hedgehogs for carriage of *T*. *erinacei*, because ethical constraints precluded us from collecting appropriate tissues (skin scrapings, hair and spines) for fungal culture, which leaves some important questions unanswered. For example, it remains unclear whether penicillin-producing *T*. *erinacei* isolates are present throughout Europe and whether there is a link between their distribution and the geographical range of *mecC*-MRSA.

In conclusion, we describe the ecological and evolutionary mechanisms that led to the emergence of methicillin resistance in the pre-antibiotic era, possibly as a co-evolutionary adaptation of *S*. *aureus* to colonization of dermatophyte-infected hedgehogs. These results underscore the importance of taking a broad One Health perspective on antibiotic resistance that recognises the role of natural selection in wild animals and the connectivity of natural, agricultural and human ecosystems in the evolution and spread of antibiotic-resistant pathogens.

## Methods

### Hedgehog survey

The aim was to collect hedgehog samples covering the geographical range of hedgehogs in Europe and New Zealand. Personnel at 16 wildlife rescue centres in ten European countries and two wildlife rescue centres in New Zealand were instructed to obtain samples from the nasal area, skin and feet of hedgehogs kept in separate enclosures using FLOQSwabs (Copan). Swabs were stored in liquid Amies medium at ambient temperature and sent to the National Reference Laboratory for Antimicrobial Resistance at Statens Serum Institut in Denmark or the Department of Veterinary Medicine at University of Cambridge in the UK immediately after sample collection. For each swab, a loopful (10 µl) of liquid Amies was inoculated into 5 ml Mueller–Hinton broth (Oxoid) supplemented with 6.5% NaCl and incubated overnight at 35–37 °C. A loopful (10 µl) of enrichment broth was then streaked on a Brilliance MRSA 2 (Oxoid) agar plate followed by incubation at 35–37 °C for 24 h. One presumptive MRSA colony from each plate was subcultured on a blood agar plate at 35–37 °C for 24 h and archived at −80 °C. We also screened all MRSA-negative samples for the presence of MSSA isolates by streaking a loopful (10 µl) of enrichment broth on a SaSelect (Bio-Rad) and Brilliance Staph 24 (Oxoid) agar plate followed by incubation at 35–37 °C for 24 h. One presumptive *S*. *aureus* colony from each plate was subcultured on a blood agar plate at 35–37 °C for 24 h and archived at −80 °C.

The hedgehog survey was conducted by staff members of the wildlife rescue centres who collected swab samples in connection with routine checks. In accordance with the Animal Welfare Act 1999 administered by the New Zealand Ministry for Primary Industries and Directive 2010/63/EU of the European Parliament and of the Council of 22 September 2010 on the protection of animals used for scientific purposes, no ethical approval was required as sample collection did not cause pain, suffering, distress or lasting harm equivalent to, or higher than, that caused by the introduction of a needle in accordance with good veterinary practice or deprived the animal of usual care. Ethical review was undertaken at the Department of Veterinary Medicine, University of Cambridge (CR76).

### Bacterial isolates and whole-genome sequencing

A list of the 1,157 isolates used in this study is provided in Supplementary Table 1, including all of the *mecC*-MRSA (*n* = 222) and MSSA (*n* = 22) isolates identified in the hedgehog survey described above and an European collection of *mecC*-MRSA (*n* = 786) and MSSA (*n* = 127) isolates belonging to CC130, CC425 and CC1943, which constitute the most successful *mecC*-MRSA clones in Europe^10,11,25^. Most of the Danish isolates originated from two nationwide collections of *mecC*-MRSA and MSSA isolates recovered from hedgehogs and humans. The Danish collection of hedgehog isolates included 114 *mecC*-MRSA isolates collected from 188 hedgehogs in a previous study^8^. We re-examined the 74 MRSA-negative hedgehog samples for the presence of MSSA isolates using the method described above, which led to the inclusion of two MSSA isolates belonging to CC130 (*n* = 1) and CC425 (*n* = 1) as well as six *mecC*-MRSA isolates belonging to CC130 (*n* = 3) and CC1943 (*n* = 3) that were missed in the original screening. The Danish collection of human isolates comprised 334 *mecC*-MRSA and 2 MSSA isolates that were collected from colonized or infected individuals between 1975 and 2016 as part of the national MRSA and *S*. *aureus* bacteraemia surveillance programmes. The remaining CC130, CC425 and CC1943 isolates were selected to represent the known geographical distribution (mainly western and central Europe) and host repertoire (mainly humans, cattle, sheep, goats and wild animals) of each clone. Whole-genome sequencing of the 1,157 isolates was performed on different Illumina platforms at the Wellcome Sanger Institute or at Statens Serum Institut. Short-read sequence data are available in the European Nucleotide Archive/NCBI Sequence Read Archive under BioProject IDs PRJEB15105, PRJEB21015, PRJEB2655, PRJEB2755, PRJEB2756, PRJEB28206, PRJEB3174, PRJEB32898, PRJNA596428 and PRJEB43456 and the genome accession numbers are provided in Supplementary Table 1.

### Sequence analyses

Draft genomes were de novo assembled using SPAdes (v.3.15)^46^. Multilocus sequence typing (MLST) was performed by comparing the draft genomes with the *S*. *aureus* MLST database^47^. We used the *scn* gene (which encodes staphylococcal complement inhibitor-A and an indicator of the IEC-1 element^48^) in *S*. *aureus* strain Newman (GenBank: NC_009641) and the *vwb*_SaPI_ gene in SaPIbov4 (GenBank: HM211303) as queries in BLASTN searches against the draft genomes, setting length match to 0.9 and similarity match to 0.7. These parameters were chosen to account for allelic diversity of the *vwb* genes located on SaPIs (sharing 76–100% nucleotide identities with each other)^49^ while effectively excluding *scn* homologues located outside the IEC-1 element (sharing 48–61% nucleotide identities with *scn*)^50^. Contigs with hits for *vwb* were analysed using PHASTER^51^ to ascertain that they were located on SaPIs. ARGs were detected by mapping sequence reads against the ResFinder database^52^ using the *k*-mer alignment (KMA) tool (v.1.3)^53^, setting both length match and similarity match to 0.9.

### Phylogenetic analyses of *S*. *aureus* CC130, CC425 and CC1943

All 991 *mecC*-MRSA and 136 MSSA isolates belonging to CC130, CC425 and CC1943 were included. Mapping of sequence reads and SNP calling were performed using NASP (v.1.0)^54^ as follows: (1) sequence reads were mapped against the reference genome of *mecC*-MRSA CC425 isolate LGA251 (GenBank: NC_017349) with the Burrows–Wheeler Alignment tool^55^; (2) SNP calling was achieved using the GATK Unified Genotyper^56,57^, setting depth of coverage and unambiguously base calls to ≥10× and ≥90%, respectively, and ignoring insertions and deletions; and (3) SNPs contained in repeats were excluded using NUCmer^58,59^.

Unrooted maximum-likelihood phylogenetic trees were built from core-genome SNP alignments with PhyML (v.3.0)^60,61^ under the HKY85 substitution model after applying NNI moves to improve the BIONJ starting tree. Putative recombinogenic regions were detected and a recombination-corrected phylogeny was built with ClonalFrameML (v.1.12)^62^. Time-resolved phylogenies, in which the date of each node is estimated, were constructed with BactDating (v.1.0)^63^ using an additive uncorrelated relaxed clock model^64^. Convergence and mixing of the Markov Chain Monte Carlo chains were determined using the R package coda^65^. The time-resolved phylogenies were rooted as inferred with BactDating (v.1.0)^63^ to maximize the posterior probability of the tree. Treedater (v.0.5)^66^ was also applied for the same purpose and found to give similar estimates for the dating of nodes (Supplementary Figs. 6–11 and Source Data of Fig. 3).

The phylogenetic relationships between the type XI SCC*mec* elements were investigated in a separate analysis. A SNP alignment was generated from precalled SNPs from the 991 *mecC*-MRSA isolates. SNPs located outside the type XI SCC*mec* element (corresponding to nucleotide positions 34,403 through 63,839 in *mecC*-MRSA CC425 isolate LGA251) were manually removed, and the remaining SNPs were used to construct an unrooted maximum-likelihood phylogenetic tree using PhyML (v.3.0)^60,61^ under the HKY85 substitution model after applying NNI moves to improve the BIONJ starting tree. Putative recombinogenic regions were detected and a recombination-corrected phylogeny was built using ClonalFrameML (v.1.12)^62^. The tips were manually mapped onto the CC130, CC425 and CC1943 phylogenies, and vice versa, to identify monophyletic *mecC*-MRSA lineages harbouring orthologous type XI SCC*mec* elements.

### Genome sequencing and analysis of the *T*. *erinacei* type strain IMI 101051

DNA extracted from the *T*. *erinacei* type strain IMI 101051 was used to prepare a DNA library in accordance with the Nextera XT DNA Library Prep Guide (Illumina) and sequenced on a MiSeq platform (Illumina) with 2 × 251 bp using a MiSeq Reagent Kit v2 (Illumina). Short-read sequencing data were submitted to the European Nucleotide Archive under BioProject PRJEB43453. A draft genome was de novo assembled using SPAdes (v.3.15)^46^. We used the translated protein sequences of the *P*. *chrysogenum pcbAB*, *pcbC* and *penDE* genes and the *A*. *chrysogenum cefD1*, *cefD2*, *cefEF* and *cefG* genes (Extended Data Table 1) as queries in TBLASTN searches against the draft genome. Hits were used as queries in BLASTP searches against the UniProtKB/Swiss-Prot protein database. Bidirectional best hits with a TBLASTN and BLASTP value of less than 1 × 10^−100^ in both directions were considered as orthologous gene pairs.

### In vitro antibiotic production by the *T*. *erinacei* type strain IMI 101051

In vitro antibiotic production by the *T*. *erinacei* type strain IMI 101051 was determined using a method modified from Smith and Marples^15^. In brief, the strain was grown on a Sabouraud dextrose agar plate (Oxoid) for 7 days at 30 °C. The culture was removed using distilled water and a 100-µl suspension was placed in 5 ml Sabouraud dextrose broth (SDB) (Sigma-Aldrich) followed by incubation at 30 °C with shaking at 200 rpm for 3 days. The culture was placed on a Miracloth mesh (Calbiochem) in a 13-cm petri dish containing 35 ml SDB. The plate was incubated at 30 °C for 7 days, after which the mesh containing the fungal mass was placed in a 2-l conical flask containing 75 ml SDB supplemented with 2% glucose. The flask was incubated at 30 °C with shaking at 200 rpm for 7 days. The broth was replaced with fresh 5-strength SDB, and the flask was incubated at 30 °C for another 6 days. The culture medium was transferred into two 50-ml Falcon tubes containing 3 ml Diaion HP-20 resin (Supelco) slurried in 3 ml distilled water. The tubes were placed on a rotator for 1 h, after which the beads were allowed to settle. The supernatant was discarded and antibiotics were eluted from the resin with 9 ml acetone. The eluate was placed on a rotator for 30 min and centrifuged briefly to pellet the resin. The supernatant was transferred to 2-ml Eppendorf tubes, which were placed in a rotary evaporator at 30 °C. This process was repeated until all of the eluate had evaporated to dryness. To prepare bacterial inocula, *S*. *aureus* strains were individually grown overnight on blood agar plates (Oxoid) at 37 °C. Colonies were suspended in phosphate-buffered saline (PBS) to a 0.5 McFarland standard, diluted 1:10 in PBS, and streaked evenly on the surface of a Iso-Sensitest agar plate (Oxoid) using a sterile cotton-wool swab. Dried fungal culture extracts were suspended in 200 µl distilled water. Sterile paper discs were impregnated with 10 µl solution and placed on the agar plates. In vitro antibiotic production was assessed by measuring the inhibition zones after overnight incubation at 35 °C.

### Metabolic profiling by LC–MS

Dried fungal culture extracts were suspended in 50% methanol to a concentration of 100 mg ml^−1^, diluted 1:10 in pure methanol and analysed using LC–MS. Metabolic profiles were obtained on a Vanquish UHPLC system (Thermo Fisher Scientific) coupled to a Vanquish diode array detector (Thermo Fisher Scientific) and an Orbitrap Fusion Tribrid high-resolution tandem mass spectrometer (Thermo Fisher Scientific). Chromatographic separation of 5 µl fungal culture extracts was performed on a Luna C18 column (3 μm, 3 × 150 mm) (Phenomenex) using a linear mobile phase gradient from 0% methanol, 90% water and 10% acetonitrile containing 1% (v/v) formic acid to 90% methanol, 0% water and 10% acetonitrile containing 1% (v/v) formic acid over 20 min at a flow rate of 400 μl min^−1^. Ultraviolet data were recorded between 210 nm and 550 nm. For comparative purposes, a pure standard of penicillin G (Sigma-Aldrich) was prepared at a final concentration of 9.4 μg ml^−1^ and analysed using LC–MS.

Mass spectra were obtained in the positive and negative ionization modes using the full scan and data-dependent MS^2^ and MS^3^ acquisition modes. Full scan total ion current chromatograms were obtained over the range of 125–1,800 *m*/*z* using a spray voltage of +3.5 kV and −2.5 kV for the positive and negative ionization modes, respectively. Three different scan events were recorded for the data-dependent acquisition modes as follows: (1) MS^2^ of the most intense ion in the full scan acquisition mode; (2) MS^3^ of the most intense ion in the MS^2^ spectra; and (3) MS^3^ of the second-most intense ion in the MS^2^ spectra. Additional parameters for the MS included the following: full scan resolution set to 60,000 (full-width at half-maximum, FWHM), capillary temperature set to 350 °C, ion transfer tube temperature set to 325 °C, RF lens set to 50%, automatic gain control target set to 4.0 × 10^5^ (full scan) or 1.0 × 10^4^ (MS^2^ and MS^3^), intensity threshold set to 1.0 × 10^4^, collision-induced dissociation energy set to 35 eV, activation *Q* set to 0.25 and isolation window set to 4 *m*/*z*. Nitrogen was used as the drying, nebulizer and fragmentation gas.

### Molecular networking analysis of LC–MS data

A molecular network was created using the Global Natural Products Social Molecular Networking (GNPS) workflow^67^. Chromatographic raw data in the positive ionization mode were transformed to the mzXML format using the ProteoWizard command-line utility msConvert^68^. The data were then filtered by removing all MS^2^ fragment ions within ±17 Da of the precursor *m*/*z* value. The precursor ion mass and MS^2^ fragment ion tolerances were set to 2.0 Da and 0.5 Da, respectively, to enable more comprehensive comparisons with the GNPS database. MS^2^ spectra were filtered by choosing the top six fragment ions in the ±50-Da window throughout the spectrum. A network was then created considering a cosine score above 0.7 and more than six matched peaks to link different nodes through edges. Edges between two nodes were kept in the network if each of the nodes appeared in each other’s respective top ten most similar nodes. Finally, the maximum size of a molecular family was set to 100. The spectra in the network were then searched against spectral libraries in GNPS, filtering the library spectra in the same manner as the input data. All matches between network spectra and library spectra were required to have a score above 0.7 and at least six matched peaks.

### Statistics

Statistical analyses were performed using GraphPad Prism (v.8.3) (GraphPad Software). We used two-tailed paired Student’s *t*-tests to compare inhibition zones of each mutant to the corresponding *mecC*-MRSA wild-type strain and a two-tailed Wilcoxon matched-pairs signed rank test to compare population structures of *mecC*-MRSA at the lineage level.

### Reporting summary

Further information on research design is available in the Nature Research Reporting Summary linked to this paper.

## Online content

Any methods, additional references, Nature Research reporting summaries, source data, extended data, supplementary information, acknowledgements, peer review information; details of author contributions and competing interests; and statements of data and code availability are available at 10.1038/s41586-021-04265-w.

### Supplementary information

Supplementary Information Supplementary Figs. 1–11 and their accompanying legends.

Reporting Summary

Supplementary Table 1 Information about the 1,157 *S*. *aureus* isolates used in this study.

Supplementary Data 1–10 Supplementary Data 1–10 and their accompanying legends.

### Source data

Source Data Fig. 1

Source Data Fig. 2

Source Data Fig. 3

Source Data Fig. 4

Source Data Extended Data Fig. 1

Source Data Extended Data Fig. 4

Source Data Extended Data Fig. 6

Source Data Extended Data Fig. 7

Source Data Extended Data Fig. 9

## Figures and Tables

**Extended Data Table 1:**
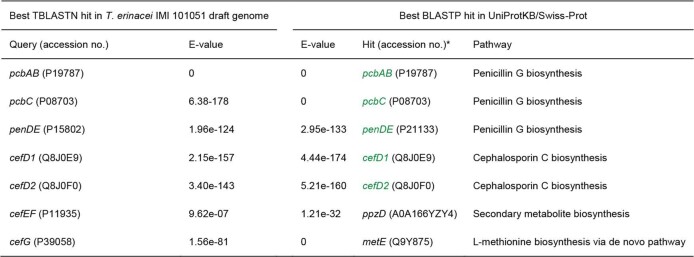
Penicillin biosynthetic genes in *T*. *erinacei* IMI 101051

*The in silico identified putative *T*. *erinacei* penicillin G and cephalosporin C biosynthetic genes are shown in green

## Data Availability

LC–MS data in mzXML format and molecular networking results are available at the MassIVE repository under identifier MSV000087335. *S*. *aureus* short-read sequence data have been deposited in the European Nucleotide Archive/NCBI Sequence Read Archive under BioProject IDs PRJEB15105, PRJEB21015, PRJEB2655, PRJEB2755, PRJEB2756, PRJEB28206, PRJEB3174, PRJEB32898, PRJNA596428 and PRJEB43456 and the genome accession numbers are provided in Supplementary Table 1. *T*. *erinacei* type strain IMI 101051 short-read sequence data have been deposited in the European Nucleotide Archive under BioProject PRJEB43453. Tree and genealogy files in Newick format are provided in Supplementary Data 1–10. Source data are provided with this paper.

## References

[1] Davies J, Davies D (2010). Origins and evolution of antibiotic resistance. Microbiol. Mol. Biol. Rev..

[2] European Centre for Disease Prevention and Control, European Medicines Agencies. *The Bacterial Challenge: Time to React. A Call to Narrow the Gap Between Multidrug-Resistant Bacteria in the EU and the Development of New Antibacterial Agents* https://ecdc.europa.eu/sites/portal/files/media/en/publications/Publications/0909_TER_The_Bacterial_Challenge_Time_to_React.pdf (2009).

[3] Jevons MP (1961). “Celbenin”—resistant Staphylococci. Br. Med. J..

[4] Harkins CP (2017). Methicillin-resistant *Staphylococcus aureus* emerged long before the introduction of methicillin into clinical practice. Genome Biol..

[5] Chambers HF, DeLeo FR (2009). Waves of resistance: *Staphylococcus aureus* in the antibiotic era. Nat. Rev. Microbiol..

[6] Price LB (2012). *Staphylococcus aureus* CC398: host adaptation and emergence of methicillin resistance in livestock. mBio.

[7] *Global Priority List of Antibiotic-Resistant Bacteria to Guide Research, Discovery, and Development of New Antibiotics* http://www.who.int/medicines/publications/WHO-PPL-Short_Summary_25Feb-ET_NM_WHO.pdf?ua=1 (WHO, 2017).

[8] Rasmussen SL (2019). European hedgehogs (*Erinaceus europaeus*) as a natural reservoir of methicillin-resistant *Staphylococcus aureus* carrying *mecC* in Denmark. PLoS ONE.

[9] Bengtsson B (2017). High occurrence of *mecC*-MRSA in wild hedgehogs (*Erinaceus europaeus*) in Sweden. Vet. Microbiol..

[10] García-Álvarez L (2011). Methicillin-resistant *Staphylococcus aureus* with a novel *mecA* homologue in human and bovine populations in the UK and Denmark: a descriptive study. Lancet Infect. Dis..

[11] Paterson GK, Harrison EM, Holmes MA (2014). The emergence of *mecC* methicillin-resistant *Staphylococcus aureus*. Trends Microbiol..

[12] Marples MJ, Smith JMB (1960). The hedgehog as a source of human ringworm. Nature.

[13] English MP, Evans CD, Hewitt M, Warin RP (1962). “Hedgehog ringworm”. Br. Med. J..

[14] Smith JMB, Marples MJ (1964). A natural reservoir of penicillin-resistant strains of *Staphylococcus aureus*. Nature.

[15] Smith JMB, Marples MJ (1965). Dermatophyte lesions in the hedgehog as a reservoir of penicillin-resistant staphylococci. J. Hyg..

[16] Smith JMB (1965). *Staphylococcus aureus* strains associated with the hedgehog *Erinaceus europaeus*. J. Hyg. Camb..

[17] Morris P, English MP (1969). *Trichophyton mentagrophytes* var. *erinacei* in British hedgehogs. Sabouraudia.

[18] Le Barzic C (2021). Detection and control of dermatophytosis in wild European hedgehogs (Erinaceus europaeus) admitted to a French wildlife rehabilitation centre. J. Fungi.

[19] Dube F, Söderlund R, Salomonsson ML, Troell K, Börjesson S (2021). Benzylpenicillin-producing *Trichophyton erinacei* and methicillin resistant *Staphylococcus aureus* carrying the *mecC* gene on European hedgehogs: a pilot-study. BMC Microbiol..

[20] Hewitt G (2000). The genetic legacy of the Quaternary ice ages. Nature.

[21] Brockie RE (1975). Distribution and abundance of the hedgehog (*Erinaceus europaeus*) L. in New Zealand, 1869–1973. N. Z. J. Zool..

[22] van den Berg MA (2008). Genome sequencing and analysis of the filamentous fungus *Penicillium chrysogenum*. Nat. Biotechnol..

[23] Ullán RV, Campoy S, Casqueiro J, Fernández FJ, Martín JF (2007). Deacetylcephalosporin C production in *Penicillium chrysogenum* by expression of the isopenicillin N epimerization, ring expansion, and acetylation genes. Chem. Biol..

[24] Kitano K (1976). A novel penicillin produced by strains of the genus. Paecilomyces. J. Ferment. Technol..

[25] Petersen A (2013). Epidemiology of methicillin-resistant *Staphylococcus aureus* carrying the novel *mecC* gene in Denmark corroborates a zoonotic reservoir with transmission to humans. Clin. Microbiol. Infect..

[26] Richardson EJ (2018). Gene exchange drives the ecological success of a multi-host bacterial pathogen. Nat. Ecol. Evol..

[27] Holden MTG (2013). A genomic portrait of the emergence, evolution, and global spread of a methicillin-resistant *Staphylococcus aureus* pandemic. Genome Res..

[28] Strauß L (2017). Origin, evolution, and global transmission of community-acquired *Staphylococcus aureus* ST8. Proc. Natl Acad. Sci. USA.

[29] Nübel U (2008). Frequent emergence and limited geographic dispersal of methicillin-resistant *Staphylococcus aureus*. Proc. Natl Acad. Sci. USA.

[30] Rasmussen SL, Nielsen JL, Jones OR, Berg TB, Pertoldi C (2020). Genetic structure of the European hedgehog (*Erinaceus europaeus*) in Denmark. PLoS ONE.

[31] Hansen JE (2019). LA-MRSA CC398 in dairy cattle and veal calf farms indicates spillover from pig production. Front. Microbiol..

[32] Eriksson J, Espinosa-Gongora C, Stamphøj I, Larsen AR, Guardabassi L (2013). Carriage frequency, diversity and methicillin resistance of in Danish small ruminants. Vet. Microbiol..

[33] Danish Integrated Antimicrobial Resistance Monitoring and Research Programme. *DANMAP 2019: Use of Antimicrobial Agents and Occurrence of Antimicrobial Resistance in Bacteria From Food Animals, Food, and Humans in DENMARK* https://www.danmap.org/-/media/Sites/danmap/Downloads/Reports/2019/DANMAP_2019.ashx?la=da&hash=AA1939EB449203EF0684440AC1477FFCE2156BA5 (2020).

[34] Veterinary Medicines Directorate. *UK Veterinary Antibiotic Resistance and Sales Surveillance Report* https://assets.publishing.service.gov.uk/government/uploads/system/uploads/attachment_data/file/950126/UK-VARSS_2019_Report__2020-TPaccessible.pdf (2020).

[35] Harrison EM (2013). Whole genome sequencing identifies zoonotic transmission of MRSA isolates with the novel *mecA* homologue *mecC*. EMBO Mol. Med..

[36] Loncaric I (2019). Characterization of *mecC* gene-carrying coagulase-negative *Staphylococcus* spp. isolated from various animals. Vet. Microbiol..

[37] Gómez P (2016). Detection of MRSA ST3061-t843-*mecC* and ST398-t011-*mecA* in white stork nestlings exposed to human residues. J. Antimicrob. Chemother..

[38] Kim C (2012). Properties of a novel PBP2A protein homolog from *Staphylococcus aureus* strain LGA251 and its contribution to the β-lactam-resistant phenotype. J. Biol. Chem..

[39] Tahlan K, Jensen SE (2013). Origins of the β-lactam rings in natural products. J. Antibiot..

[40] Pantůček R (2018). *Staphylococcus edaphicus* sp. nov. isolated in Antarctica harbors the *mecC* gene and genomic islands with a suspected role in adaptation to extreme environment. Appl. Environ. Microbiol..

[41] D’Costa VM (2011). Antibiotic resistance is ancient. Nature.

[42] Allen HK, Moe LA, Rodbumrer J, Gaarder A, Handelsman J (2009). Functional metagenomics reveals diverse beta-lactamases in a remote Alaskan soil. ISME J..

[43] Forsberg KJ (2012). The shared antibiotic resistome of soil bacteria and human pathogens. Science.

[44] Forsberg KJ (2014). Bacterial phylogeny structures soil resistomes across habitats. Nature.

[45] Coll F (2020). Definition of a genetic relatedness cutoff to exclude recent transmission of meticillin-resistant *Staphylococcus aureus*: a genomic epidemiology analysis. Lancet Microbe.

[46] Bankevich A (2012). SPAdes: a new genome assembly algorithm and its application to single-cell sequencing. J. Comput. Biol..

[47] Enright MC, Day NP, Davies CE, Peacock SJ, Spratt BG (2000). Multilocus sequence typing for characterization of methicillin-resistant and methicillin-susceptible clones of *Staphylococcus aureus*. J. Clin. Microbiol..

[48] Van Wamel WJ, Rooijakkers SH, Ruyken M, van Kessel KP, Strijp JA (2006). The innate immune modulators staphylococcal complement inhibitor and chemotaxis inhibitory protein of *Staphylococcus aureus* are located on beta-hemolysin-converting bacteriophages. J. Bacteriol..

[49] Viana, D. et al. *Adaptation of Staphylococcus aureus* to ruminant and equine hosts involved SaPI-carried variants of von Willebrand factor-binding protein. *Mol. Microbiol*. **77**, 1583–1594 (2010). 10.1111/j.1365-2958.2010.07312.x 20860091

[50] Rooijakkers SHM (2007). Staphylococcal complement inhibitor: structure and active sites. J. Immunol..

[51] Arndt D (2016). PHASTER: a better, faster version of the PHAST phage search tool. Nucleic Acids Res..

[52] Bortolaia V (2020). ResFinder 4.0 for predictions of phenotypes from genotypes. J. Antimicrob. Chemother..

[53] Clausen PTLC, Aarestrup FM, Lund O (2018). Rapid and precise alignment of raw reads against redundant database with KMA. BMC Bioinform..

[54] Sahl JW (2016). NASP: an accurate, rapid method for the identification of SNPs in WGS datasets that supports flexible input and output formats. Microb. Genom..

[55] Li H, Durbin R (2009). Fast and accurate short read alignment with Burrow-Wheeler transform. Bioinformatics.

[56] McKenna A (2010). The Genome Analysis Toolkit: a MapReduce framework for analyzing next-generation DNA sequencing data. Genome Res..

[57] DePristo MA (2011). A framework for variation discovery and genotyping using next-generation sequencing data. Nat. Genet..

[58] Delcher AL, Phillippy A, Carlton J, Salzberg SL (2002). Fast algorithms for large-scale genome alignment and comparison. Nucleic Acids Res..

[59] Kurz S (2004). Versatile and open software for comparing large genomes. Genome Biol..

[60] Guindon S, Gasquel O (2003). A simple, fast, and accurate algorithm to estimate large phylogenies by maximum likelihood. Syst. Biol..

[61] Guindon S (2010). New algorithms and methods to estimate maximum-likelihood phylogenies: assessing the performance of PhyML 3.0. Syst. Biol..

[62] Didelot X, Wilson DJ (2015). ClonalFrameML: efficient inference of recombination in whole bacterial genome. PLoS Comput. Biol..

[63] Didelot X (2018). Bayesian inference of ancestral dates on bacterial phylogenetic trees. Nucleic Acids Res..

[64] Didelot X, Siveroni I, Volz EM (2021). Additive uncorrelated relaxed clock models for the dating of genomic epidemiology phylogenies. Mol. Biol. Evol..

[65] Plummer M, Best N, Cowles K, Vines K (2006). CODA: convergence diagnosis and output analysis for MCMC. R News.

[66] Volz EM, Frost SD (2017). Scalable relaxed clock phylogenetic dating. Virus Evol..

[67] Wang M (2016). Sharing and community curation of mass spectrometry data with Global Natural Products Social Molecular Networking. Nat. Biotechnol..

[68] Adusumilli R, Mallick P (2017). Data conversion with ProteoWizard msConvert. Methods Mol. Biol..

