# Supplementary material for: Emergence of methicillin resistance predates the clinical use of antibiotics
Source: Nature. 2022 Jan 5;602(7895):135–41. doi: 10.1038/s41586-021-04265-w (PMC8810379; doi:10.1038/s41586-021-04265-w)
Supplement: Supplementary file 1 — Supplementary Figs. 1–11 and their accompanying legends. [file 41586_2021_4265_MOESM1_ESM.pdf]

nature portfolio

https://doi.org/10.1038/s41586-021-04265-w

Supplementary information

Emergence of methicillin resistance predates the clinical use of antibiotics

In the format provided by the
authors and unedited

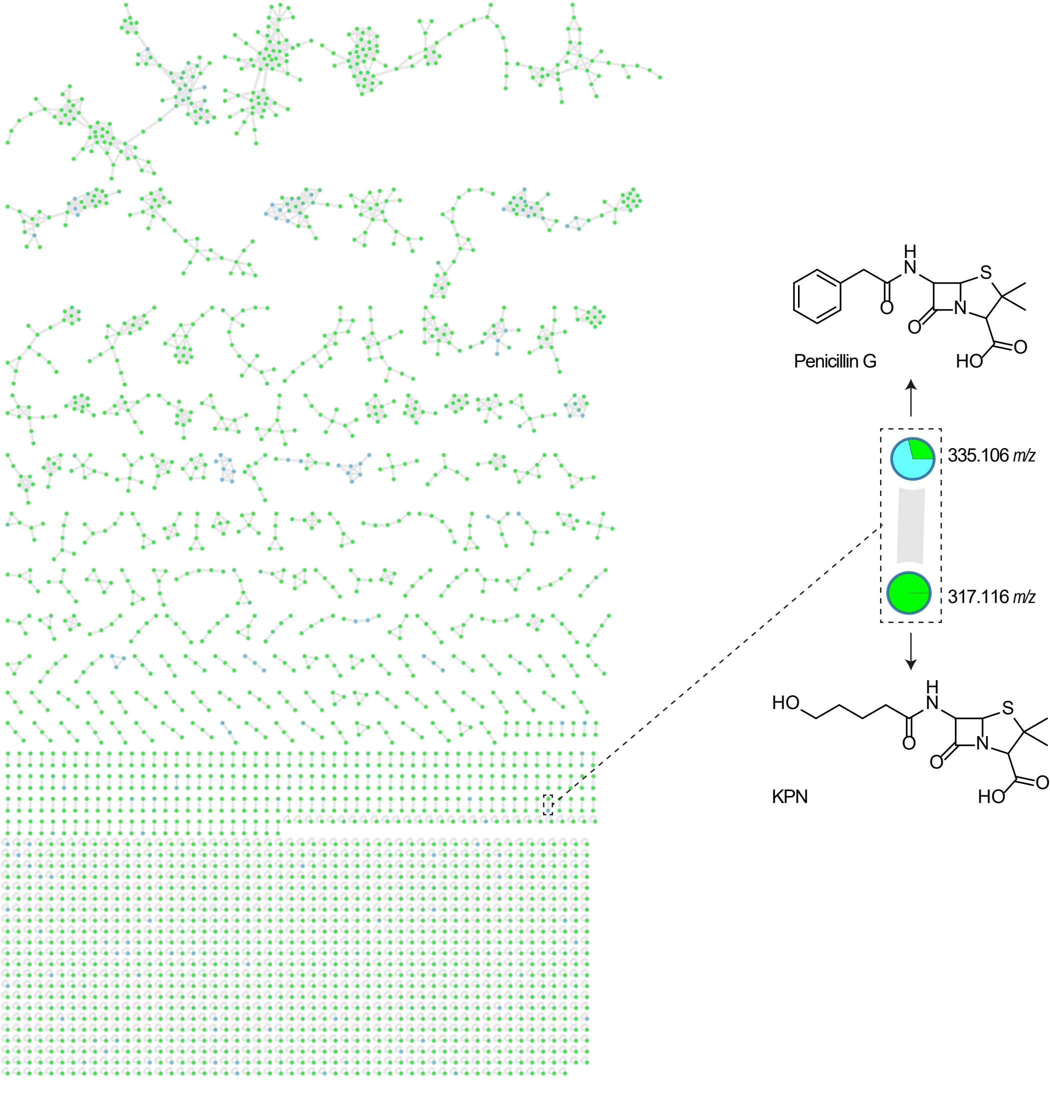

Supplementary Fig. 1 | Molecular networking analysis of LC-MS data from *T. erinacei* culture broth. Nodes within the network are represented as pie charts to visualize spectral count differences for each molecule between a pure standard of penicillin G (blue) and *T. erinacei* culture broth (green). The connected nodes corresponding to penicillin G and KPN are highlighted.

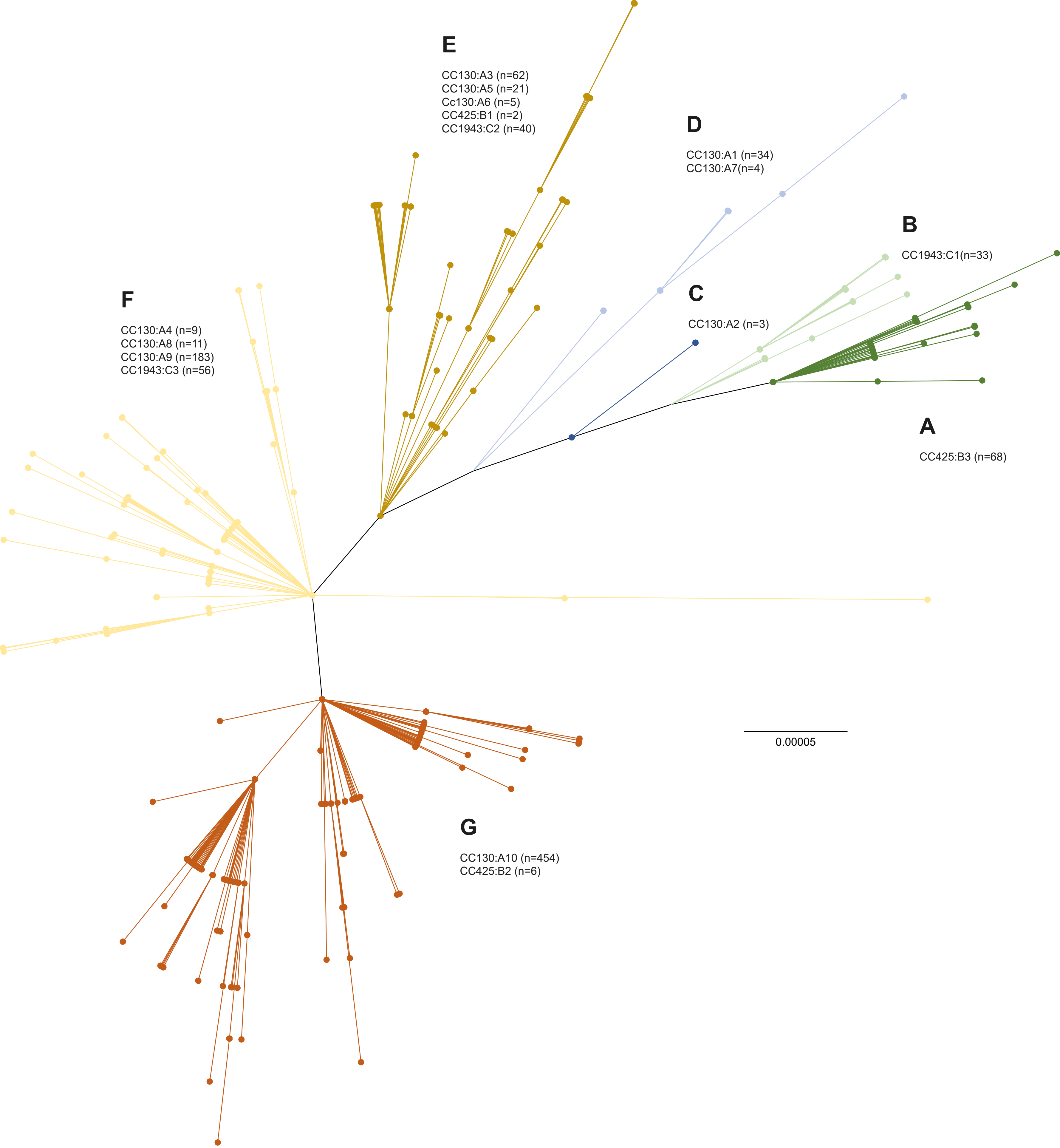

Supplementary Fig. 2 | Phylogenetic tree of the type XI SCC*mec* element from 991 *mecC*-MRSA CC130, CC425, and CC1943 isolates. The undated ClonalFrameML phylogeny was estimated for 350 high-quality core SNPs. The scale bar represents the number of nucleotide substitutions per site. The evolution of the type XI SCC*mec* elements could be traced back to seven nodes that were connected to each other on a long backbone (black branches). Each of the backbone nodes and its orthologous descendants received the same letter designation to reflect their genetic relationship (A to G). Tips are illustrated by filled circles coloured to indicate the type XI SCC*mec* variant. Manual mapping of the tips onto the CC130, CC425, and CC1943 phylogenies, and vice versa, enabled us to assign the *mecC*-MRSA isolates to 16 monophyletic lineages harbouring orthologous type XI SCC*mec* elements (Supplementary Figs. 3-5).

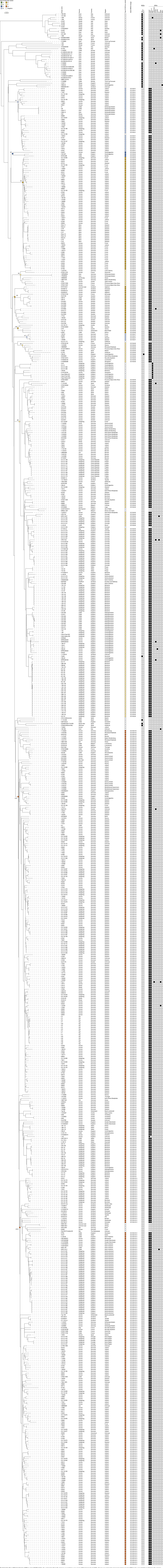

Supplementary Fig. 3 | Phylogenetic tree of 853 *S. aureus* CC130 isolates. The undated ClonalFrameML phylogeny was built from a core-genome SNP alignment (34,117 SNPs). The tree was rooted at the most recent common ancestor (MRCA) of *S. aureus* CC130 (see Methods). The scale bar represents the number of nucleotide substitutions per site. Manual mapping of the type XI SCC*mec* variants (Supplementary Fig. 2) onto the phylogeny enabled us to assign the 786 *mecC*-MRSA CC130 isolates to ten monophyletic lineages harbouring orthologous type XI SCC*mec* elements (CC130:A1 through CC130:A10). The CC130:A10 isolates forming a distinct sublineage (CC130:A10.1) are also displayed (see Results and Figs. 3 and 4). The MRCA of each lineage are illustrated by filled circles coloured to indicate the type XI SCC*mec* variant. Presence and absence of antibiotic resistance genes (ARGs) and mobile genetic elements (MGEs) encoding human- and ruminant-specific immune modulators involved in host switching events are shown as black and white boxes, respectively.

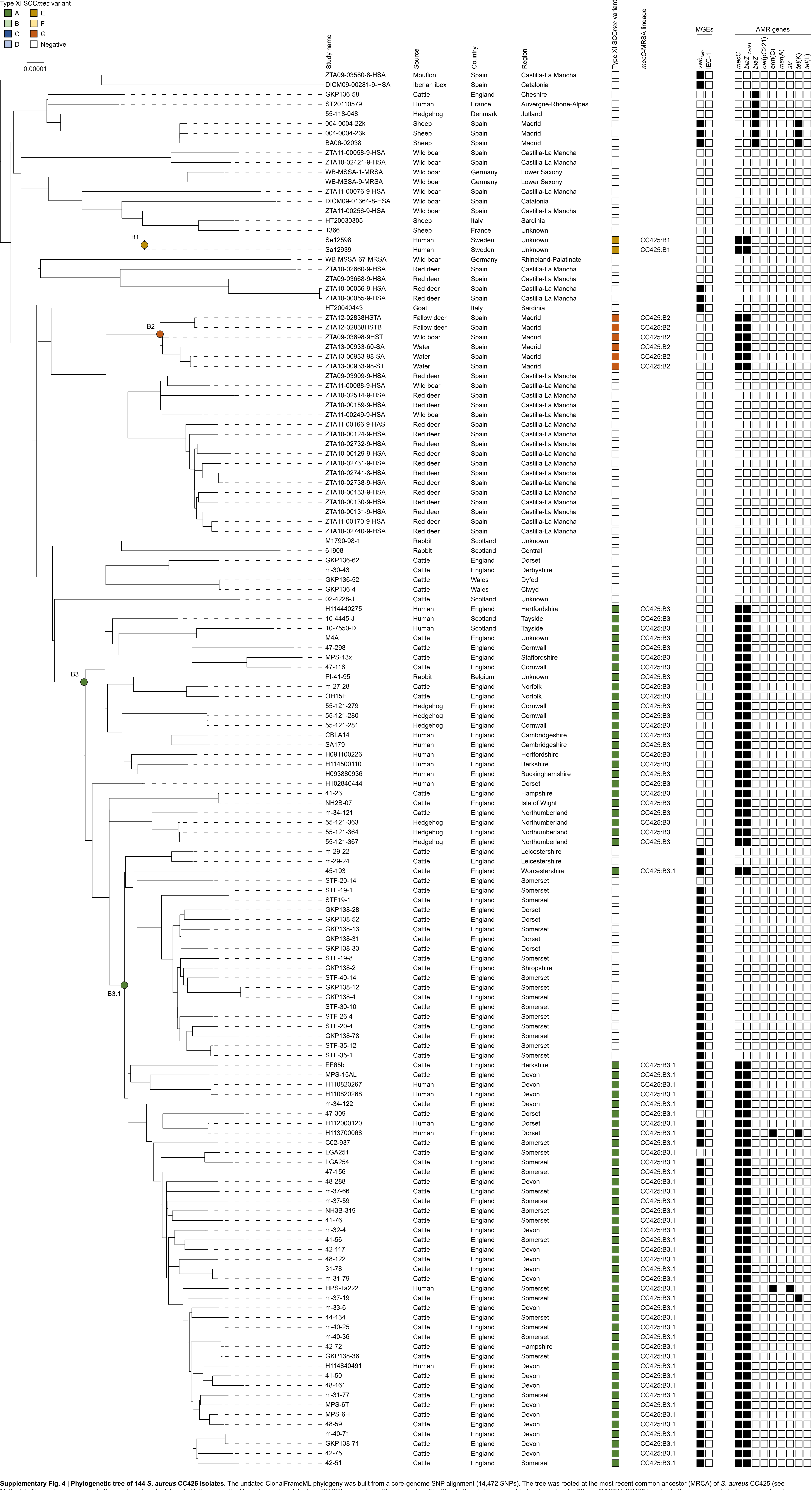

Supplementary Fig. 4 | Phylogenetic tree of 144 *S. aureus* CC425 isolates. The undated ClonalFrameML phylogeny was built from a core-genome SNP alignment (14,472 SNPs). The tree was rooted at the most recent common ancestor (MRCA) of *S. aureus* CC425 (see Methods). The scale bar represents the number of nucleotide substitutions per site. Manual mapping of the type XI SCC*mec* variants (Supplementary Fig. 2) onto the phylogeny enabled us to assign the 76 *mecC*-MRSA CC425 isolates to three monophyletic lineages harbouring orthologous type XI SCC*mec* elements (CC425:B1 through CC425:B3). The CC425:B3 isolates forming a distinct sublineage (CC425:B3.1) are also displayed (see Results and Figs. 3 and 4). The MRCA of each lineage are illustrated by filled circles coloured to indicate the type XI SCC*mec* variant. Presence and absence of antibiotic resistance genes (ARGs) and mobile genetic elements (MGEs) encoding human- and ruminant-specific immune modulators involved in host switching events are shown as black and white boxes, respectively.

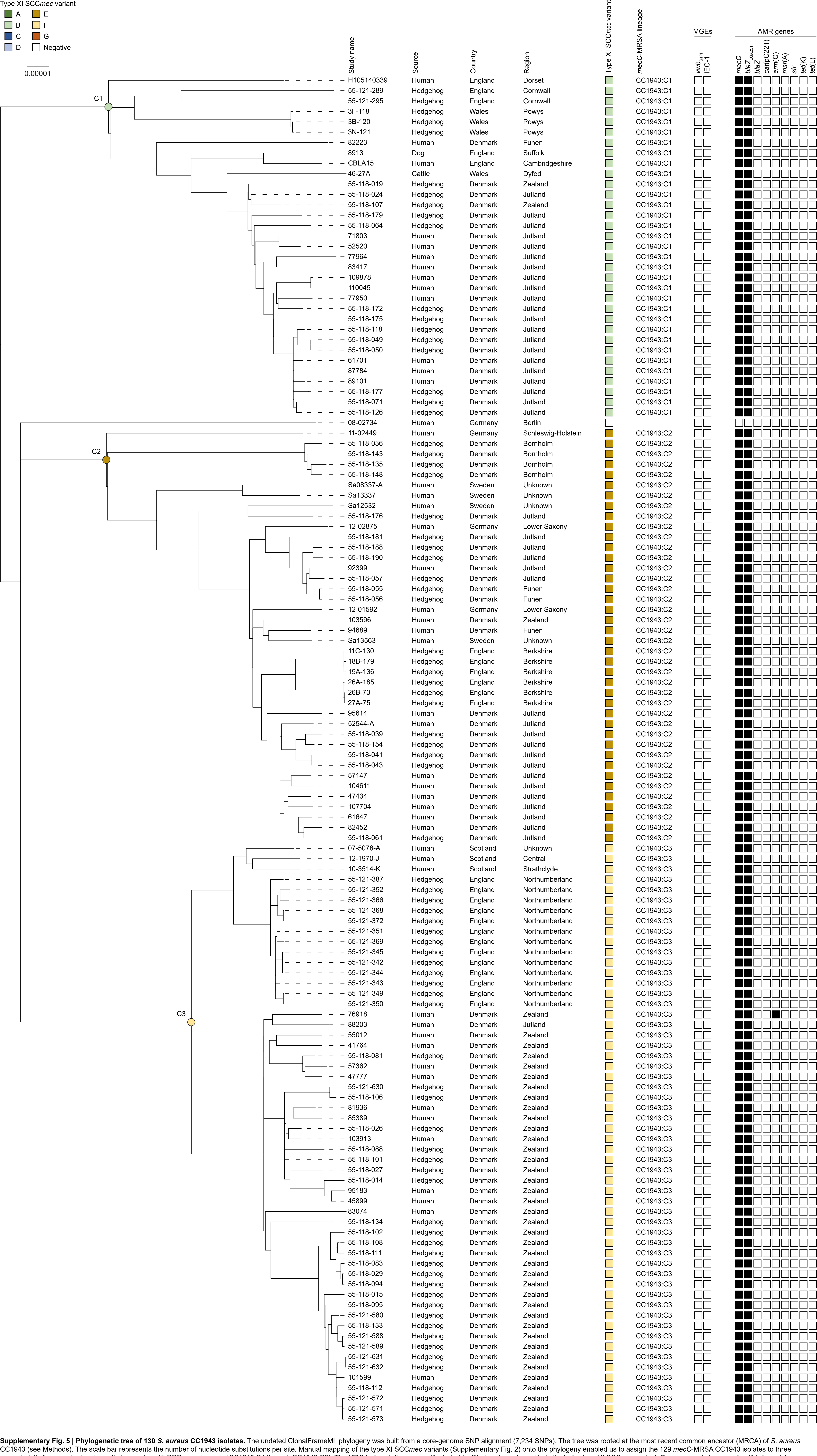

Supplementary Fig. 5 | Phylogenetic tree of 130 *S. aureus* CC1943 isolates. The undated ClonalFrameML phylogeny was built from a core-genome SNP alignment (7,234 SNPs). The tree was rooted at the most recent common ancestor (MRCA) of *S. aureus* CC1943 (see Methods). The scale bar represents the number of nucleotide substitutions per site. Manual mapping of the type XI SCC*mec* variants (Supplementary Fig. 2) onto the phylogeny enabled us to assign the 129 *mecC*-MRSA CC1943 isolates to three monophyletic lineages harbouring orthologous type XI SCC*mec* elements (CC1943:C1 through CC1943:C3). The MRCA of each lineage are illustrated by filled circles coloured to indicate the type XI SCC*mec* variant. Presence and absence of antibiotic resistance genes (ARGs) and mobile genetic elements (MGEs) encoding human- and ruminant-specific immune modulators involved in host switching events are shown as black and white boxes, respectively.

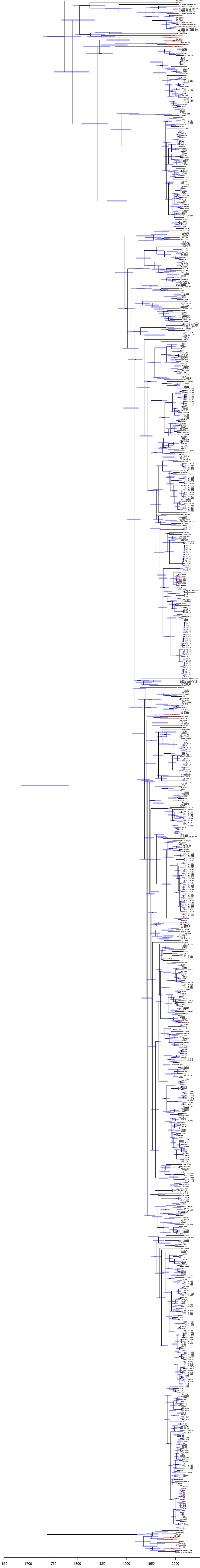

Supplementary Fig. 6 | BactDating genealogy of 853 *S. aureus* CC130 isolates. The genealogy was built from the recombination-corrected ClonalFrameML tree (Supplementary Fig. 3). Blue and red bars show the 95% confidence intervals for ancestral dates and unknown sampling dates, respectively.

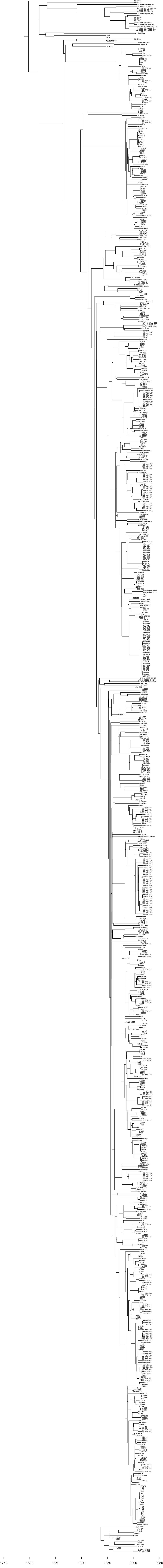

Supplementary Fig. 7 | treedater genealogy of 853 *S. aureus* CC130 isolates. The genealogy was built from the recombination-corrected ClonalFrameML tree (Supplementary Fig. 3).

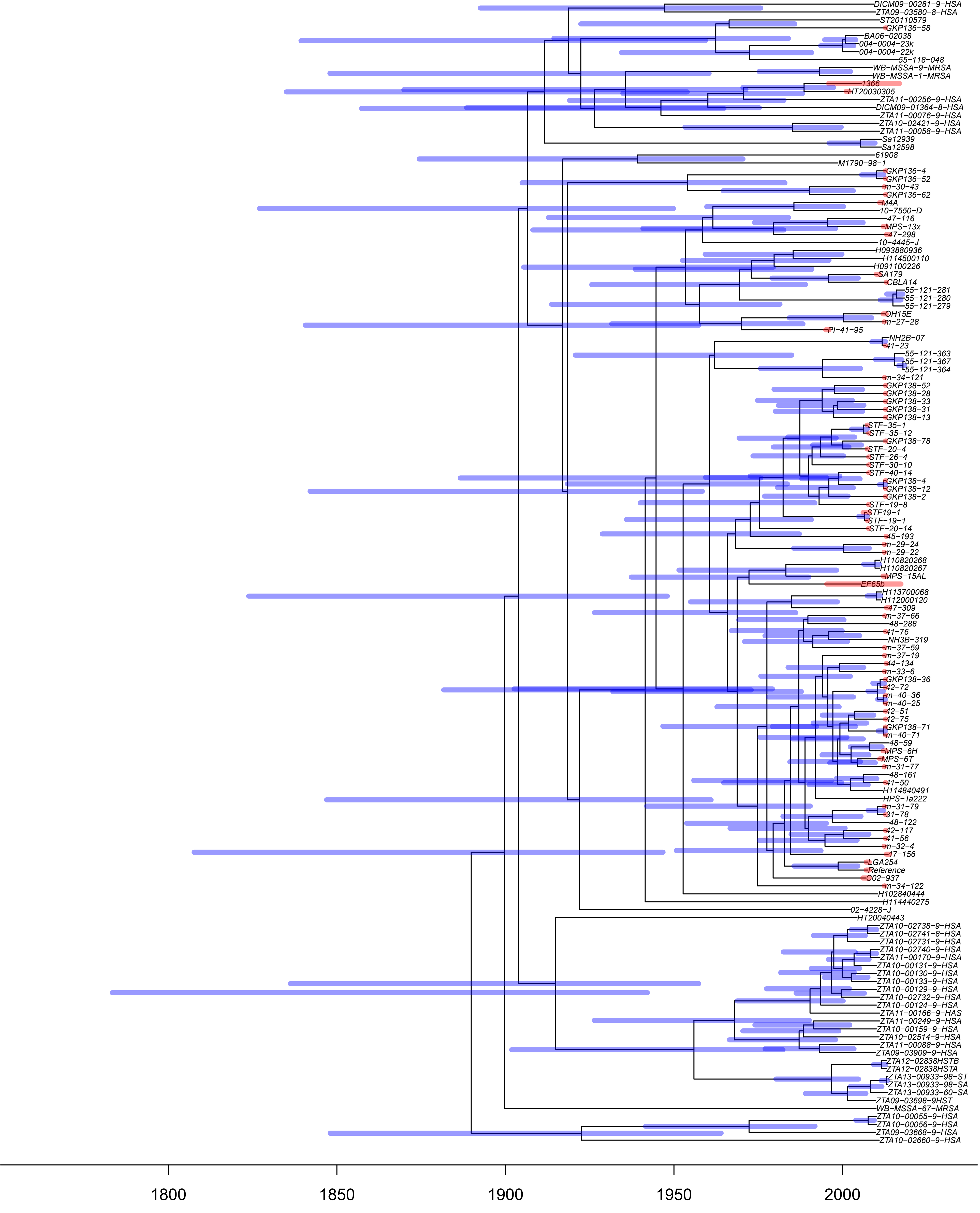

Supplementary Fig. 8 | BactDating genealogy of 144 *S. aureus* CC425 isolates. The genealogy was built from the recombination-corrected ClonalFrameML tree (Supplementary Fig. 4). Blue and red bars show the 95% confidence intervals for ancestral dates and unknown sampling dates, respectively.

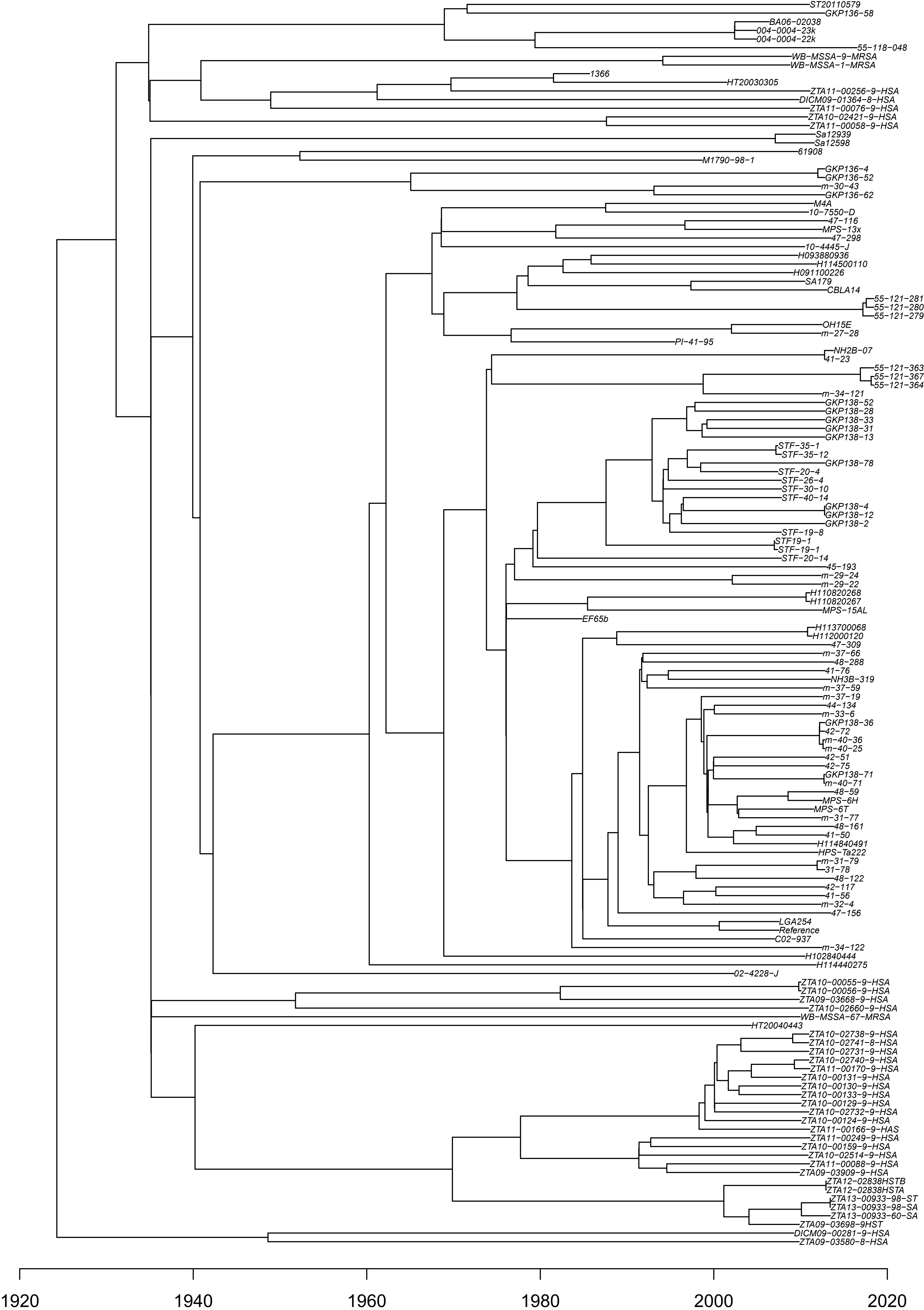

Supplementary Fig. 9 | treedater genealogy of 144 *S. aureus* CC425 isolates. The genealogy was built from the recombination-corrected ClonalFrameML tree (Supplementary Fig. 4).

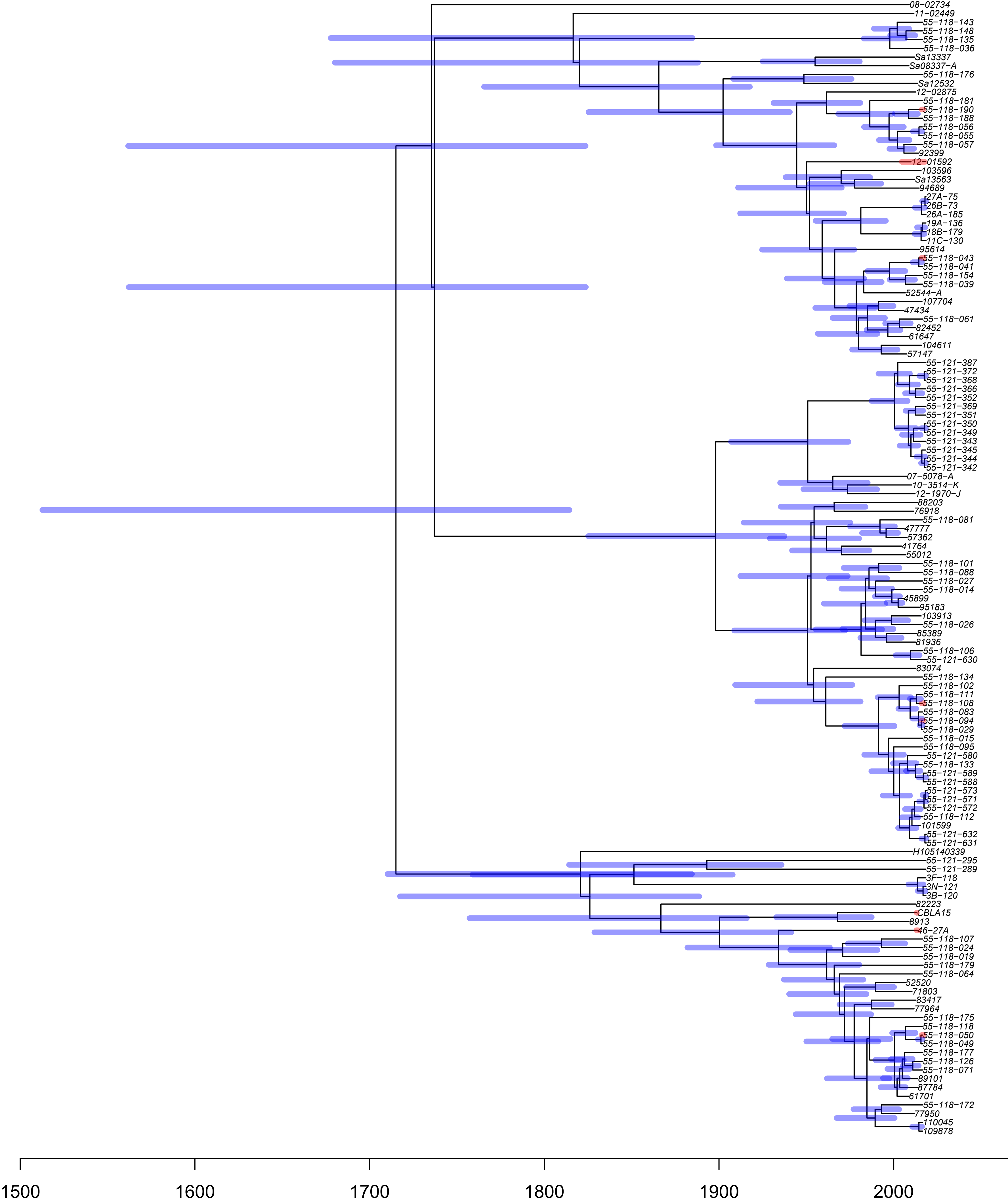

Supplementary Fig. 10 | BactDating genealogy of 130 *S. aureus* CC1943 isolates. The genealogy was built from the recombination-corrected ClonalFrameML tree (Supplementary Fig. 5). Blue and red bars show the 95% confidence intervals for ancestral dates and unknown sampling dates, respectively.

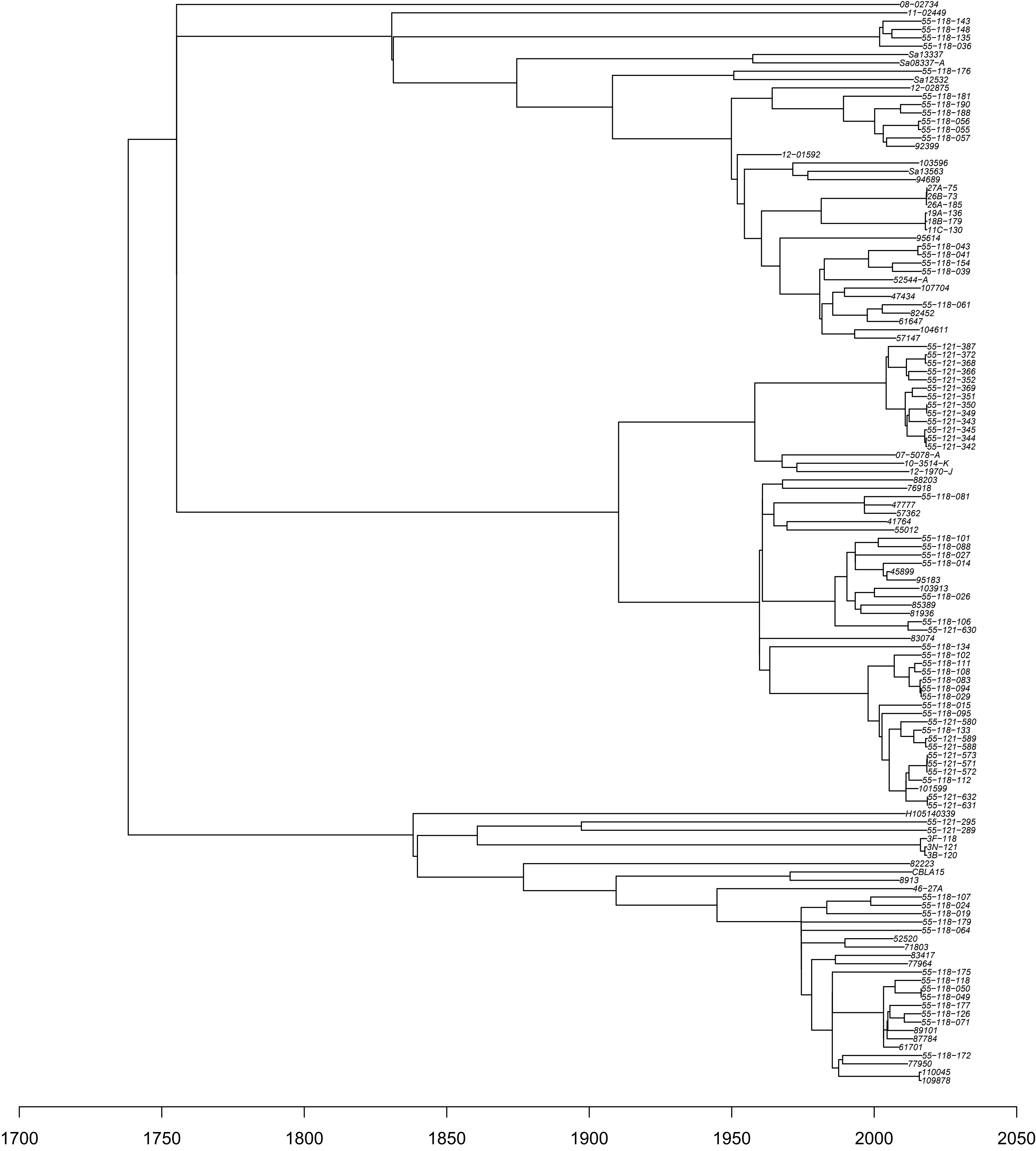

Supplementary Fig. 11 | treedater genealogy of 130 *S. aureus* CC1943 isolates. The genealogy was built from the recombination-corrected ClonalFrameML tree (Supplementary Fig. 5).
